# The *Taxu*s genome provides insights into paclitaxel biosynthesis

**DOI:** 10.1038/s41477-021-00963-5

**Published:** 2021-07-15

**Authors:** Xingyao Xiong, Junbo Gou, Qinggang Liao, Yanlin Li, Qian Zhou, Guiqi Bi, Chong Li, Ran Du, Xiaotong Wang, Tianshu Sun, Lvjun Guo, Haifei Liang, Pengjun Lu, Yaoyao Wu, Zhonghua Zhang, Dae-Kyun Ro, Yi Shang, Sanwen Huang, Jianbin Yan

**Affiliations:** 1 College of Horticulture, Hunan Agricultural University, Changsha, China; 2 Shenzhen Branch, Guangdong Laboratory for Lingnan Modern Agriculture, Shenzhen Key Laboratory of Agricultural Synthetic Biology, Genome Analysis Laboratory of the Ministry of Agriculture and Rural Affairs, Agricultural Genomics Institute at Shenzhen, Chinese Academy of Agricultural Sciences, Shenzhen, China; 3 Engineering Research Center for Horticultural Crop Germplasm Creation and New Variety Breeding, Ministry of Education, Changsha, China; 4 Peng Cheng Laboratory Artificial Intelligence Research Center No. 2, Shenzhen, China; 5 MOE Key Laboratory of Bioinformatics, Tsinghua-Peking Joint Center for Life Sciences, School of Life Sciences, Tsinghua University, Beijing, China; 6 College of Horticulture, Qingdao Agricultural University, Qingdao, China; 7 Department of Biological Sciences, University of Calgary, Calgary, Alberta Canada; 8 The AGISCAAS-YNNU Joint Academy of Potato Sciences, Yunnan Normal University, Kunming, China

**Keywords:** Plant evolution, Secondary metabolism, Jasmonic acid

## Abstract

The ancient gymnosperm genus *Taxus* is the exclusive source of the anticancer drug paclitaxel, yet no reference genome sequences are available for comprehensively elucidating the paclitaxel biosynthesis pathway. We have completed a chromosome-level genome of *Taxus chinensis* var. *mairei* with a total length of 10.23 gigabases. *Taxus* shared an ancestral whole-genome duplication with the coniferophyte lineage and underwent distinct transposon evolution. We discovered a unique physical and functional grouping of *CYP725A*s (cytochrome P450) in the *Taxus* genome for paclitaxel biosynthesis. We also identified a gene cluster for taxadiene biosynthesis, which was formed mainly by gene duplications. This study will facilitate the elucidation of paclitaxel biosynthesis and unleash the biotechnological potential of *Taxus*.

## Abstract (web summary)

A high-quality *Taxus* genome provides insight into the genome evolution of the *Taxus* genus and the biosynthesis of paclitaxel.

## Main

Taxaceae, a widespread family of non-flowering conifers with substantial economic value, contains six extant genera and over 28 species^1^. *Taxus* is the largest genus in Taxaceae, including common species such as *T. chinensis*, *T. brevifolia* and *T. baccata*, and it is mainly distributed in Asia, North America and Europe^2^. For decades, *Taxus* has served as a natural source of paclitaxel (trade name Taxol), a well-known chemotherapy agent against various cancers^3^. But plant-derived paclitaxel suffers from a short supply due to its low abundance in *Taxus*, limiting its clinical application. Multiple strategies have been employed to address supply issues^4^, and promising progress has been made in chemical^5^ and semichemical synthesis^6^, direct extraction from *Taxus* cell lines^7^, fermentation of endophytic paclitaxel-producing fungi^8^ and metabolic engineering of paclitaxel production using heterologous systems^9^.

As a tetracyclic diterpene, paclitaxel is biosynthesized by a complex metabolic pathway^10^. The paclitaxel pathway starts with geranylgeranyl diphosphate (GGPP) synthesis through the condensation of isoprenyl diphosphate and dimethylallyl diphosphate^11^. GGPP is then cyclized by taxadiene synthetase (TS), generating a unique diterpene skeleton, taxadiene^12^. Taxadiene is subsequently decorated by a series of reactions including hydroxylation, oxidation, epoxidation, acylation and benzoylation to generate the final product via catalysis by various enzymes (for example, hydroxylase, oxidase, epoxidase, oxomutase and transferase)^13–15^. To date, over 20 enzymes have been identified in the paclitaxel biosynthetic pathway. However, several essential steps in the pathway, such as C1 hydroxylation, C9 oxygenation and oxetane formation, remain to be clarified. Moreover, studies have shown that jasmonates, gibberellin, auxin and ethylene are involved in the regulation of paclitaxel biosynthesis to maintain a delicate balance between growth and defence in *Taxus*^16–18^. Several transcription factors (TFs), including AP2/ERF, WRKY, MYC and MYB, have been found to regulate the expression of paclitaxel biosynthetic genes^19–21^. However, the comprehensive regulatory mechanisms underlying the growth–defence trade-off are still poorly understood.

A complete *Taxus* genome sequence can provide valuable bioinformatic and genetic resources to understand paclitaxel biosynthesis and regulatory mechanisms in depth, but the size and complexity of the *Taxus* genome (2C-value, 22.3–24.3 picograms) have hindered its de novo draft genome assembly to date^22^. Here, we have successfully assembled the *Taxus* genome, and we present a reference-grade genome sequence of *T. chinensis* var. *mairei* containing 10.23 gigabases (Gb) of data with contig N50 of 2.44 megabases (Mb), 9.86 Gb of which was assigned to 12 pseudochromosomes. We demonstrate that the CYP725A (cytochrome P450) genes, closely related to paclitaxel biosynthesis, have evolved independently in a unique physical and functional grouping in the *Taxus* genome. Moreover, we have uncovered a gene cluster for taxadiene biosynthesis that contains a new type of TS. These results contribute to our understanding of the biological and evolutionary questions regarding paclitaxel biosynthesis and provide insights into the genome structure and organization of gymnosperms.

## Results

### *Taxus* genome sequencing, assembly and annotation

**Fig. 1:**
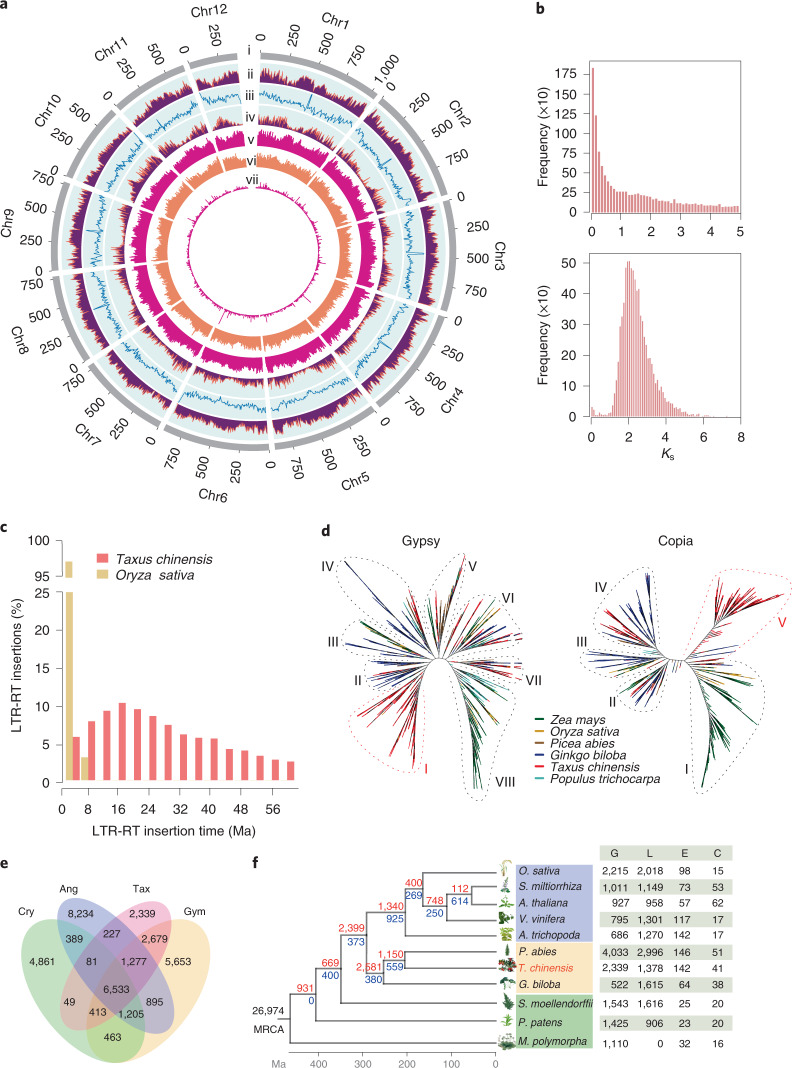
Genomic features of *T. chinensis* var. *mairei*. **a**, Genomic landscape of the 12 assembled pseudochromosomes. Track i represents the length of the pseudochromosomes (Mb); ii–iv represent repeat element density, GC content and distribution of gene density, respectively; and v–vii show the distribution of Ty3/Gypsy, Ty1/Copia and unknown LTRs, respectively. These metrics are calculated in 5 Mb windows. **b**, WGD analysis based on the substitution rate distribution of paralogues. Top, histogram of the *K*_s_ distribution from *Taxus* paralogues based on an all-to-all blast to total genes. Bottom, *K*_s_ distribution of paralogues based on syntenic analysis. The *K*_s_ values were calculated using the YN model in *KaKs*_calculator. **c**, Expansions and diverse sets of LTR elements in the *Taxus* genome. The histogram shows distributions of insertion times calculated for LTRs in *Taxus* and rice, using mutation rates (per base year) of 7.3 × 10^−10^ for *Taxus* and 1.8 × 10^−8^ for rice. The LTR-retrotransposon (LTR-RT) insertions of *T. chinensis* var. *mairei* and *Oryza sativa* are shown as columns in different colours. **d**, Heuristic maximum likelihood trees of Ty3/Gypsy (shown as Gypsy) and Ty1/Copia (shown as Copia) from six plant species. The two trees were constructed from amino acid sequence similarities within the reverse transcriptase domains of Gypsy and Copia from six plant species. Gypsy elements are divided into eight families (I–VIII), and Copia contains five families (I–V). The representative plants are shown as coloured lines. **e**, Venn diagram for orthologous protein-coding gene clusters in cryptogam (Cry), angiosperm (Ang), gymnosperm (Gym) and *T. chinensis* var. *mairei* (Tax). The cryptogams include *M. polymorpha*, *Physcomitrella* *patens* subsp. *patens* and *Selaginella* *moellendorffii*. The angiosperms include *Amborella trichopoda*, *V. vinifera*, *Arabidopsis thaliana*, *Salvia miltiorrhiza* and *O. sativa*. The gymnosperms include *Picea abies* and *Ginkgo biloba*. The number in each sector of the diagram represents the total number of genes across the four comparisons. **f**, Evolution analysis of gene families in *Taxus* and selected plants. The red numbers on the branches of the phylogenetic tree indicate the number of expanded gene families, and the blue numbers refer to the number of constricted gene families. The supposed most recent common ancestor (MRCA) contains 26,974 gene families. G, L, E and C in the table at right represent the number of gains, losses, expansions and constrictions in the gene families among 11 plant species.

**Extended Data Fig. 1:**
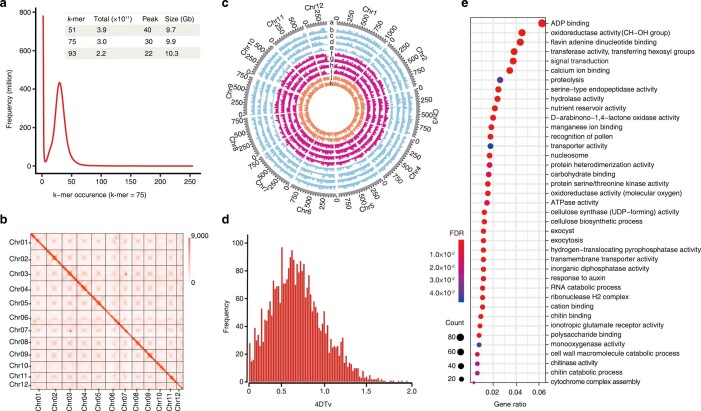
The Taxus genomic features to complement Fig. 1. a, Genome size estimation of *T. chinensis* var. *mairei* based on *k*-mer distribution. The X-axis represents the occurrence of *k*-mers, and the Y-axis represents the frequency. The *k*-mer values for different genome sizes are shown in the inner table. b, Genome-wide all-by-all Hi-C interaction. The heat map shows Hi-C interactions under a resolution of 2 Mb. Darker red pixels indicate higher contact probabilities. The number on the scale bar indicates the number of links after logarithmic analysis. c, Genomic landscape of the twelve pseudochromosomes. Track a represents the length of the pseudochromosomes (Mb); b, c, d, and e show the expression of tissue-specific genes in the bark of stem, root, strobili and leaf from the male Taxus plant, respectively; f, g, h, and i show the expression of tissue-specific genes in the bark of stem, root, strobilus and leaf from the female Taxus plant, respectively; j and k display high- and low- producing paclitaxel cell lines, respectively. d, Whole genome duplication (WGD) analysis based on the substitution rate distribution of paralogs. The 4DTv values of paralogs were calculated using *KaKs*_calculator with the YN model. The X-axis is the value of fourfold synonymous third-codon transversions (4DTv) for paralogous pairs in the Taxus genome, and the Y-axis represents the frequency. e, Gene Ontology (GO) enrichment for gene families with significant expansion. GO enrichment analysis of a subset of 142 gene families with significant expansion (*p* < 0.05); FDRs were adjusted for multiple testing. The size and color of dots indicate the number of genes and false discovery rate (FDR), respectively. The X-axis represents the gene ratio, and the GO terms are listed on the Y axis.

To build a chromosome-level genome assembly of *Taxus*, genomic DNA was extracted from endosperm calli. The endosperm of *T. chinensis* var. *mairei* seeds with haploid chromosomes was used to culture the callus, as it could prevent the influence of heterozygous elements in the genome assembly. *K*-mer analysis showed that the genome size of *T. chinensis* var. *mairei* was approximately 10 Gb (Extended Data Fig. 1a), which is consistent with the results from the flow cytometry tests^23^. A de novo assembly of the *Taxus* genome was achieved by PacBio continuous long reads (318.05 Gb) and augmented with Illumina whole-genome sequencing reads (693.73 Gb) (Supplementary Table 1). After the application of high-throughput/resolution chromosome conformation capture (Hi-C) (Supplementary Table 2), 9.86 Gb of sequence data could be assigned to 12 pseudochromosomes (Extended Data Fig. 1b and Supplementary Table 3), which covered 96.28% of the genome (Supplementary Table 4). We finally obtained the genome sequence with a total length of 10.23 Gb and a contig N50 of 2.44 Mb (Fig. 1a and Supplementary Table 4).

On the basis of the genomic information, 42,746 protein-coding genes were further annotated by integrating transcriptome data, homologous alignments and ab initio gene models. In total, 73.02% of the genes (31,214 out of 42,746) could be supported by RNA-seq data (Extended Data Fig. 1c and Supplementary Table 5). The BUSCO analysis further demonstrated that 1,052 of the 1,614 core genes were complete, showing relatively high completeness of the assembled genome in gymnosperms (Supplementary Table 6). Furthermore, 36,518 coding genes, accounting for 85.43% of the total predicted genes, were assigned to functional categories with an *E*-value less than 10^−5^ (Supplementary Table 7).

### *Taxus* experienced a whole-genome duplication event in the cupressophyte clade

Given that whole-genome duplication (WGD) is a important evolutionary force contributing to the expansion of plant genome size^24^, we investigated whether *Taxus* had experienced any WGD events. We built a paralogous gene pair set by performing an all-against-all blastp search. The number of synonymous substitutions per synonymous site (*K*_s_) of paralogues was calculated using the gene pair set. As shown in Fig. 1b, the frequency of *K*_s_ values exhibited an apparent decay without a natural distribution with increasing *K*_s_ values, which indicated that no recent WGD event occurred in the *Taxus* genome. Moreover, we noticed that most of the *K*_s_ values were less than 0.8, indicating that gene duplication in combination with saturation and stochasticity effects may obscure WGD^24^. We further used MCScanX to produce 8,148 syntenic gene pairs from the all-against-all blastp data and entered them into the *K*_s_ and distance-transversion rate at fourfold degenerate sites (4DTv) calculations. The results showed two signature peaks located at 2.1 for *K*_s_ (Fig. 1b) and 0.7 for 4DTv (Extended Data Fig. 1d), suggesting the presence of an ancient WGD in *Taxus*. Together with previous studies that revealed an ancient WGD event (WGD-ζ) in the common ancestor of angiosperms and gymnosperms^24,25^, all the above results suggest that *Taxus* shared the common ancient WGD with other coniferophyte lineages.

### *Taxus* genome expansion is linked with retrotransposons

Except for the role of WGD in enlarging the *Taxus* genome size, we noticed that repetitive sequences constituted a important component of the *Taxus* genome (Supplementary Table 8). There was a total of 7.79 Gb of repetitive sequences, occupying 76.09% of the entire genome (Supplementary Table 8). Among these repetitive sequences, long terminal repeat (LTR) retrotransposons accounted for the highest proportion at 52.38% (Supplementary Table 8). The insertion time analysis revealed that LTR insertion was a continuous process, and approximately 40% of the insertions occurred 8 to 24 million years ago (Ma) (Fig. 1c). This feature of continuous insertion in the *Taxus* genome was distinctly different from that in the rice genome, where almost 95% of LTR insertions occurred within the last 5 million years^26^. Considering that LTR insertion in Norway spruce and ginkgo mainly occurred 12–24 and 16–24 Ma^23,25^, the continuous insertion of LTRs might be a common phenomenon in gymnosperms.

To further explore the evolution of LTR in *Taxus*, we analysed the phylogenies of LTR retrotransposons in a few representative gymnosperm and angiosperm plants. Amino acid sequence similarities within the reverse transcriptase domain of the Ty3/Gypsy retrotransposons (Gypsy) and Ty1/Copia retrotransposons (Copia) were used to construct phylogenetic trees. As shown in Fig. 1d, the Gypsy superfamily members of the gymnosperms ginkgo and *Picea* were distributed in families II–VII, while those of angiosperms mainly belonged to family VIII. In contrast, *Taxus* Gypsy elements not only were distributed in families II–VIII but also evolved a highly species-specific family (family I), suggesting the expansion of specific Gypsy elements after *Taxus* speciation. Similarly, the unique expansion phenomenon in *Taxus* was also observed in the phylogenies of the Copia superfamily (Fig. 1d). Moreover, family V consisted of only *Taxus* LTRs in the Copia phylogenetic tree displaying a *Taxus*-specific amplification burst. In addition, *Taxus* was distributed in family IV, where the gymnosperms ginkgo and *Picea* were located, and families I–III also contained angiosperms, suggesting that *Taxus* LTRs were placed in a unique position compared with other selected species. These results suggest that the Gypsy and Copia superfamilies of *Taxus* have undergone a relatively unique evolutionary pattern, especially the specific Gypsy family I and Copia family V.

### Evolution of gene families and elevated secondary metabolism in *Taxus*

To understand the context of metabolic networks during *Taxus* evolution, we compared orthologous genes between *Taxus* and selected gymnosperms, angiosperms and cryptogams (Fig. 1e). In the 35,298 identified orthologous gene families (Supplementary Table 9), we found that 6,533 gene families were shared by the selected species, illustrating their evolutionary conservation (Fig. 1e). Compared with the selected species, 2,339 gene families were exclusive to *Taxus* (Fig. 1e). In addition, 1,378 gene families experienced loss, while 142 and 41 families underwent expansion and contraction in *Taxus*, respectively (Fig. 1f).

*Taxus* contains 9,747 unique genes (Fig. 1e and Supplementary Table 10), many of which are enriched in the biosynthesis of specialized metabolites, including terpenes, phenylpropanoids and flavones (Supplementary Table 11). For instance, 57 gene families were annotated to be cytochrome P450 (CYP450) gene families (Supplementary Table 10). Gene expansion analysis demonstrated that 979 genes were enriched in ADP binding, oxidoreductase activity, flavin adenine dinucleotide binding, transferase activity and signal transduction, among other functions (Extended Data Fig. 1e and Supplementary Table 12), with eight gene families being associated with CYP450 (Supplementary Table 13). Pfam functional analysis further showed that the *Taxus* genes were enriched in CYP450 gene families (PF00067.22, *P* < 0.01) and TFs (PF13837.6, *P* < 0.01; and PF00847.20, *P* < 0.01) (Supplementary Table 14). KEGG analysis indicated that the gained and expanded gene families were enriched in a total of 36 and 41 KEGG pathways, respectively, including one phenylpropanoid (ko00940) and three terpenoid metabolic pathways (ko00900, ko00130 and ko00902) (Supplementary Tables 11 and 15).

### Evolution and genomic organization of *Taxus* CYP450s

**Fig. 2:**
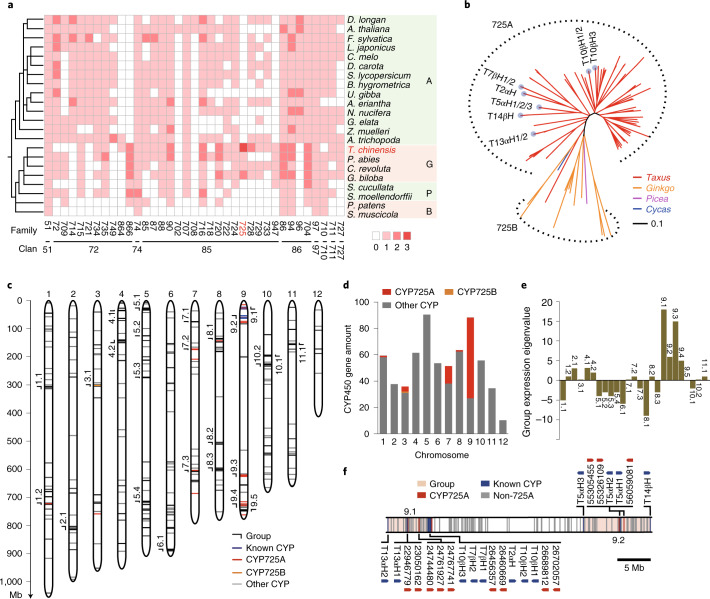
Evolution and genomic architecture of *Taxus* CYP450s. **a**, Phylogenomic analysis of the non-A-type CYP450s in the representative plant species. A, angiosperms; G, gymnosperms; P, pteridophytes; B, bryophytes. The colour of each block is based on the number of genes in each family, and 0, 1, 2 and 3 indicate that this number ranges from 0, 1–10, 10–50 and 50–100 genes, respectively. **b**, Phylogenetic analysis of the CYP725 subfamily in *T. chinensis* var. *mairei* (*Taxus*), *Ginkgo biloba* (*Ginkgo*), *Picea abies* (*Picea*) and *C. revolute* (*Cycas*). The dotted outline shows the gene spheres of the CYP725A and CYP725B subfamilies. The light blue dots on the ends of the phylogenetic branches represent the known paclitaxel pathway CYP725A genes and their homologues. The neighbour-joining tree was constructed by Interactive Tree Of Life (iTOL) software. The evolutionary distances were analysed by the *p*-distance method, and the branch lengths were scaled by the bar. **c**, Distribution of CYP450 genes on the 12 pseudochromosomes in *Taxus*. Each short line on the pseudochromosomes represents a CYP450 gene. CYP725As, CYP725Bs and the other CYP450s are marked by red, orange and grey lines, respectively. The known CYP450s in the paclitaxel biosynthesis pathway (known CYP) are shown in blue. The CYP450 groups (≥7 CYP450 genes and ≤5.26 Mb of gene spacing between two adjacent CYP450s) are labelled outside of the corresponding positions on the pseudochromosomes. **d**, Histogram of the number of CYP450 genes on each pseudochromosome. The CYP725 genes (shown in red and orange) were mainly distributed on pseudochromosome 9, while the other CYPs (shown in grey) were distributed randomly on 12 pseudochromosomes. The *y* axis represents the number of CYP450 genes. **e**, Group-based gene expression profiles in response to methyl jasmonate (MeJA) treatment. RNA sequencing analysis was performed with the low-paclitaxel-yielding cell line (LC) treated with 100 μM MeJA for 4 h. The expression of the gene group was calculated by the sum of the expression levels of each CYP450, and each upregulated and downregulated CYP450 was calculated as 1 and −1, respectively, on the basis of their reads per kilobase per million reads values. **f**, Map of CYP725As located in groups 9.1 and 9.2. The ranges of the gene groups on pseudochromosome 9 are marked in pink. CYP725As and the other genes are marked by red and grey vertical lines, respectively. The known CYP450s in the paclitaxel biosynthesis pathway (known CYP) are shown in blue. The arrows show gene orientations.

**Extended Data Fig. 2:**
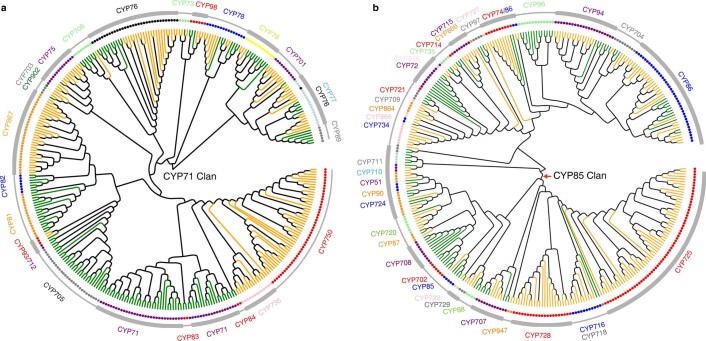
Phylogenic analysis of A-type and non-A-type CYP450 families. **a**, Phylogenic analysis of A-type CYP450 families. The green and orange branches indicate the sequences from Arabidopsis and *T. chinensis* var. *mairei*, respectively. The dots represent CYP450 genes. The outermost circle indicates the CYP450 gene family. **b**, Phylogenic analysis of non-A-type CYP450 families. The green and orange branches indicate the sequences from *Arabidopsis* and *T. chinensis* var. *mairei*, respectively. The dots represent CYP450 genes. The outermost circle indicates the CYP450 gene family.

**Extended Data Fig. 3:**
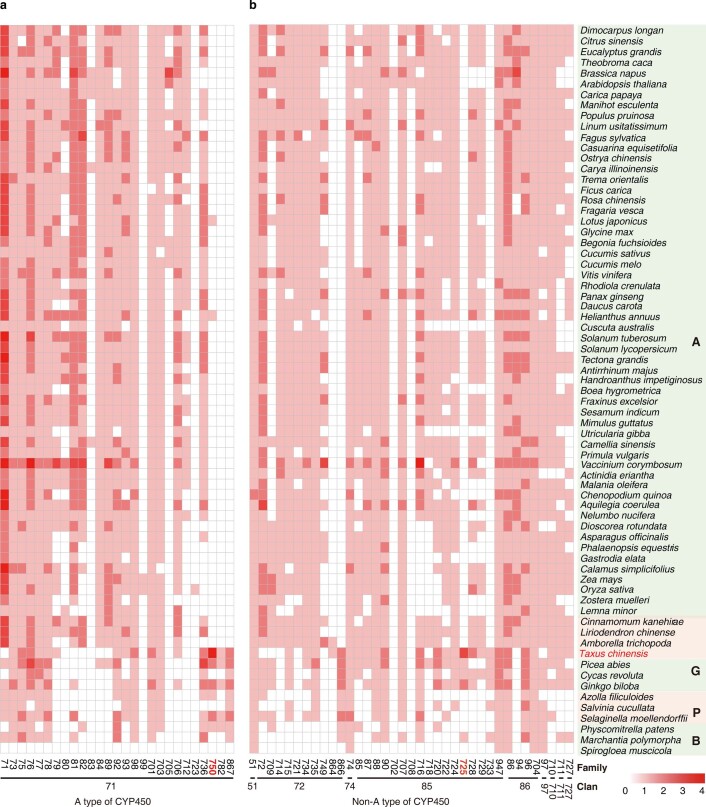
Heat map of the number of CYP450 genes in 69 representative plant species. Each CYP450 gene family of A-type (**a**) and non-A-type (**b**) is represented as a square, with the red color representing the number of genes in the corresponding family. The depth of the red color is divided into five levels, namely, 0, 1, 2, 3, and 4, which correspond to 0, 1–10, 10–50, 50–100, and more than 100 genes, respectively. The family or clan name of CYP450 genes is marked below the heat map. A, Angiosperms; G, Gymnosperms; P, Pteridophytes; and B, Bryophytes.

Given that CYP450s participate in almost half of the enzymatic reactions in paclitaxel biosynthesis^27^, we analysed *Taxus* CYP450 families and identified 649 CYP450 genes from the present genome using the reported HMM model (PF00067). These CYP450s can be divided into two catalogues: A-type and non-A-type. The A-type CYP450s included only the CYP71 clan, which consisted of 17 families and 325 genes (Extended Data Fig. 2a and Supplementary Table 16), while the non-A-type CYP450s contained 12 clans that were composed of 27 families and 324 genes (Extended Data Fig. 2b and Supplementary Table 16). Phylogenomic analyses showed that the CYP750 and CYP725 families were obviously expanded in *Taxus* compared with 68 other representative species, which covered Zygnematophyceae and Sapindaceae (Fig. 2a, Extended Data Fig. 3a,b and Supplementary Table 17). The CYP750 family was reported to participate in the biosynthesis of thujone monoterpene, which is involved in defence responses (for example, resistance against herbivore feeding)^28^, while CYP725 genes were known to contribute to paclitaxel biosynthesis^29^. Phylogenetic analysis of these CYP725 genes further showed that they could be categorized into the CYP725A and CYP725B subfamilies (Fig. 2b). The CYP725A subfamily (a total of 79 genes) exhibited specificity to *Taxus*, whereas the CYP725B subfamily was universal in gymnosperm plants (including *Picea*, *Cycas*, *Ginkgo* and *Taxus*) (Fig. 2b), which suggested that CYP725A underwent independent evolution in *Taxus*. Considering that all the previously defined CYP450 genes in the paclitaxel pathway belong to the CYP725A subfamily, these results suggest that the expansion of the CYP725A subfamily played vital roles in the evolution of paclitaxel biosynthesis in *Taxus*.

**Extended Data Fig. 4:**
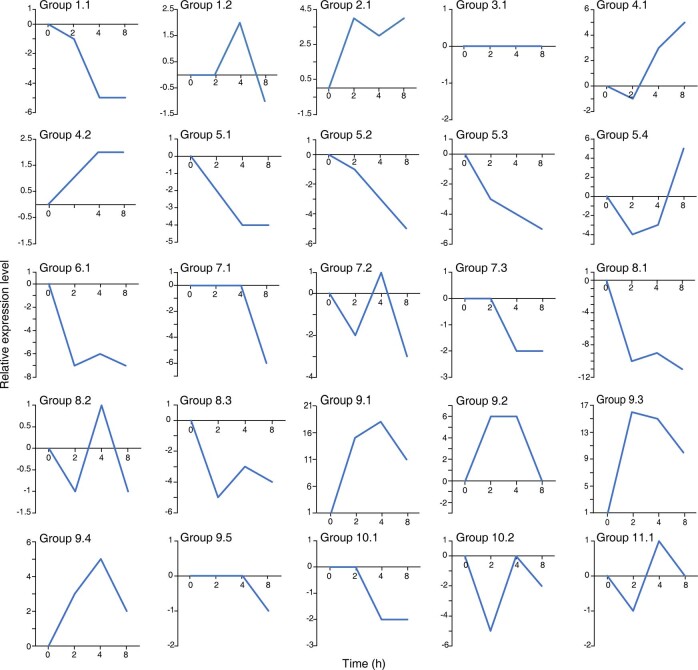
Gene expression in response to MeJA treatment in the Taxus cell line. Group-based gene expression profiles in response to MeJA treatment. RNA sequencing analysis was performed with the Taxus cell line treated with 100 μM MeJA or 0.5% EtOH solution for 0, 2, 4, and 8 h. The expression of the gene group was calculated by summing the expression levels of each CYP450. Each upregulated and downregulated CYP450 was calculated as 1 and −1, respectively, based on their FPKM (Fragments Per Kilobase of transcript per Million mapped reads) values.

We noticed that most CYP725A genes (74.68%) were located on pseudochromosome 9 (Fig. 2c,d), exhibiting a distinct non-uniform distribution. Gene location analysis further revealed that the *Taxus* CYP450 genes were not distributed randomly but tended to organize into different gene groups, 25 of which were detected in the genome (Fig. 2c). We found that nearly all these groups, except groups 1.2 and 5.1, contained gene members from no more than three CYP450 families, and 11 groups had only one CYP450 family (Supplementary Table 18), suggesting that the grouping of CYP450 genes on the genome had an obvious family aggregation pattern. Furthermore, as an essential phytohormone in the biosynthesis of secondary metabolites^30^, jasmonate is closely related to the expression regulation of CYP450 genes in *Taxus* (Fig. 2e). Under jasmonate treatment, eight groups showed an obviously increased expression level, and ten groups showed clear inhibition of gene expression (Fig. 2e, Extended Data Fig. 4 and Supplementary Table 18). These results suggest that the CYP450s in the majority of groups were coexpressed under jasmonate treatment, implying that the grouping of CYP450 genes had some coordination of physiological functions.

The gene expression levels of four gene groups (group 9.1–9.4) on pseudochromosome 9 were upregulated most prominently in the presence of jasmonate (Fig. 2e). More interestingly, groups 9.1 and 9.2 contained all known CYP725A subfamily genes related to paclitaxel biosynthesis and 12 undefined CYP725As (Fig. 2f). The expression profiles of these two groups of CYP725A genes showed that 88% of CYP725As were highly expressed in roots, 79% of CYP725As were highly expressed in the high-paclitaxel-yielding cell line (HC) and 88% of CYP725As were upregulated after jasmonate treatment (Supplementary Table 19), which is consistent with the results on the increased level of baccatin III and paclitaxel in the *Taxus* cell line under jasmonate treatment (Supplementary Fig. 1). These results suggest that the two groups are likely to contain most of the paclitaxel pathway genes that arose during *Taxus* evolution.

### Taxadiene biosynthetic genes are arranged in gene clusters

**Fig. 3:**
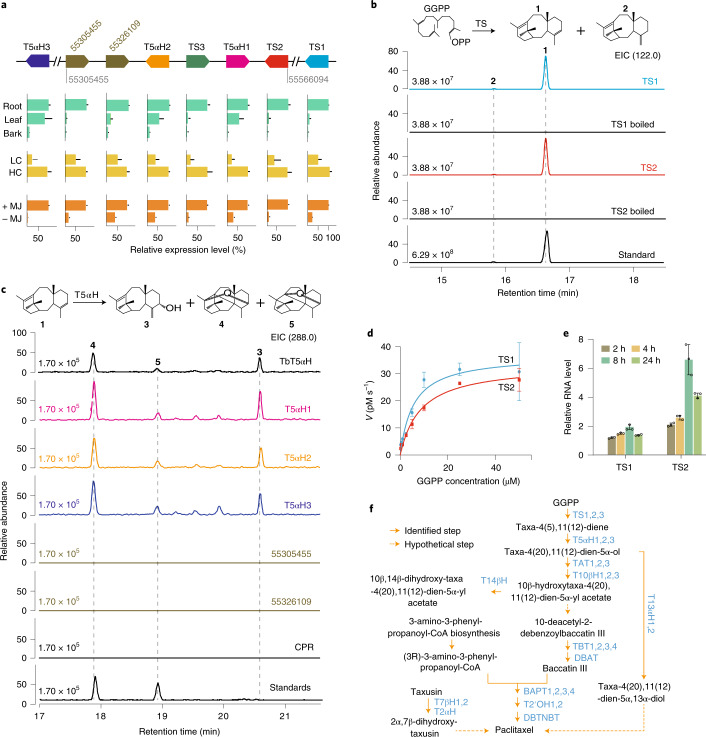
Functional identification of the paclitaxel biosynthesis gene cluster. **a**, Genomic architecture and expression pattern of the taxadiene cluster. The arrows indicate the relative positions and directions of the genes in the cluster. Here, 55305455 and 55566094 indicate the starting and ending positions of the cluster on pseudochromosome 9. The two unknown CYP725A genes are represented by their gene starting positions (55326109 and 55305455). TS1 and T5αH3 are located at 72105619–72109598 and 49866845–49868629 bp on chromosome 9, respectively. The relative expression levels of taxadiene cluster genes in *Taxus* are based on their reads per kilobase per million reads values. The expression levels of genes with high sequence similarity were distinguished on the basis of sequencing read counts of the exons that include different bases, and adjusting the alignment threshold to no mismatch. RNA-seq datasets are from roots, leaves and bark of male plants (shown in green); two *T. chinensis* var. *mairei* half-sib cell lines, HC and LC (shown in yellow); and MeJA-treated LC (+MJ) and MeJA-untreated LC (−MJ) (shown in orange). The data are shown as means ± s.d. (*n* = 3 biologically independent samples). **b**, Analysis of TS activity in vitro. The purified recombinant TS1-His and TS2-His were incubated with the substrate GGPP overnight at 32 °C. The reaction products were analysed by GC–MS. TS catalyses GGPP to produce a major product (taxa-4(5),11(12)-diene (**1**)) and a minor product (taxa-4(20),11(12)-diene (**2**)), while boiled TSs have no TS activity. *m/z* 122 is a characteristic ion of taxadienes. The taxadiene confirmed by NMR analysis was used as a reference standard (Standard). EIC, extracted ion chromatograms; OPP, pyrophosphoric acid. **c**, Analysis of the activity of T5αH and two unknown CYP725As in vitro. The in vitro enzyme assay was carried out with the purified taxadiene substrate and yeast microsomes, each including one of the six CYPs (T5αH1, T5αH2, T5αH3, TbT5αH, 55326109 or 55305455) and CPR. T5αH1/2/3 can produce three oxygenated taxadiene products (5(12)-oxa-3(11)-cyclotaxane (**3**), 5(11)-oxa-3(11)-cyclotaxane (**4**) and taxa-4(20),11(12)-dien-5*α*-ol (**5**)), whereas no catalytic compounds were observed for 55326109, 55305455 and CPR. *T. brevifolia* taxadiene 5-α-hydroxylase (TbT5αH), shown to have taxadiene 5-α-hydroxylase activity, was used as a positive control. **d**, Kinetic evaluation of GGPP oxidation catalysed by TS1 (blue circles) and TS2 (red rectangles). The *x* axis indicates the substrate GGPP concentration, while the *y* axis shows the velocity (V) of enzymatic reaction. *K*_m_ = 5.5 ± 1.6 μM (TS1), *K*_m_ = 8.6 ± 1.5 μM (TS2), *k*_cat_ = 1705 s^−1^ (TS1) and *k*_cat_ = 3282 s^−1^ (TS2). The data are shown as means ± s.d. (*n* = 3 biologically independent samples). **e**, Quantitative real-time PCR analysis of the transcription levels of TS1 and TS2 in the *Taxus* cell line LC treated with 100 μM MeJA for the indicated times. The relative gene expression levels are represented as the average fold change (2^*−ΔΔCt*^). The *Taxus* actin 1 gene (*7G702435613*) was used as an internal reference. The data are shown as means ± s.d. (*n* = 3 biologically independent samples). **f**, Biosynthesis pathway of paclitaxel in *T. chinensis* var. *mairei*. The solid arrows indicate the identified steps in the paclitaxel pathway, whereas the dashed arrows show the hypothetical steps. The compounds in the pathway are shown in black and the catalytic enzymes are shown in blue. T5αH, taxadiene 5-α-hydroxylase; T13αH, taxane 13-α-hydroxylase; TAT, taxadien-5-α-ol O-acetyltransferase; T10βH, taxane 10-β-hydroxylase; T14βH, taxoid 14-β-hydroxylase; T2αH, taxoid 2-α-hydroxylase; T7βH, taxoid 7-β-hydroxylase; TBT, 2-α-hydroxytaxane 2-O-benzoyltransferase; DBAT, 10-deacetylbaccatinIII 10-O-acetyltransferase; BAPT, baccatin III amino phenylpropanoyl-13-O-transferase; DBTNBT, 3′-N-debenzoyl-2′-deoxytaxol N-benzoyl transferase. Source data

PlantiSMASH^31^ analysis further showed that a potential gene cluster related to terpene biosynthesis was presented in group 9.2 (Fig. 3a and Supplementary Table 20). The gene cluster contained two TS genes (*TS2* and *TS3*, sharing 99.96% nucleotide sequence identity), two T5αH genes (*T5αH1* and *T5αH2*, sharing 98.67% nucleotide sequence identity) and two unknown CYP725As (Fig. 3a, Supplementary Table 21 and Supplementary Figs. 2 and 3). Moreover, the genes in the cluster showed a highly coordinated tissue expression pattern and expression consistency in response to jasmonate treatment (Fig. 3a), suggesting that the genes could be functionally related. *TS2* and *TS3* were located adjacent to *T5αH1* and *T5αH2* (Fig. 3a), suggesting that the genes involved in the first two paclitaxel biosynthetic steps were organized by a tandem gene duplication event during *Taxus* genomic evolution. The *K*_s_ value of these duplicated genes suggested that this *TS*–*T5αH* duplication occurred approximately 1.15 Ma. In addition to the *TS* and *T5αH* genes assembled in the cluster, additional *TS* (*TS1*) and *T5αH* (*T5αH3*) genes are located downstream and upstream of the cluster, respectively (Fig. 3a). Biochemical assays further confirmed that *TS1/2* and *T5αH1/2/3* have TS activity (Fig. 3b) and taxa-4(5),11(12)-diene-5α-hydroxylase activity (Fig. 3c and Supplementary Fig. 4), respectively, demonstrating that the copied genes possessed the corresponding enzyme activities in *T. chinensis* var. *mairei*.

**Extended Data Fig. 5:**
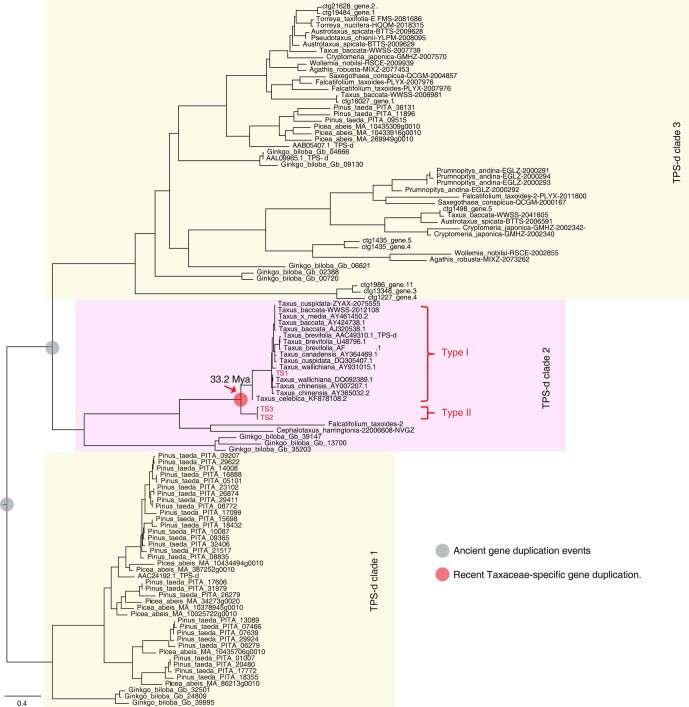
Phylogenetic analysis of trehalose-6-phosphate synthase d subfamily (TPS-d) genes from different plants. The tree is generated from amino acid sequences by the maximum-likelihood method with 100 bootstraps. Ancient gene duplication events are indicated as gray dots, while the more recent Taxaceae-specific gene duplication is shown as a red dot. The *TS1/2/3* genes in *T. chinensis* var. *mairei* are highlighted in red.

We further studied the kinetic properties of TS1 and TS2. The *K*_m_ value of TS2 was approximately 1.5 times higher than that of TS1, but the turnover number (*k*_cat_) of TS2 was nearly 2 times greater than that of TS1, indicating that TS2 might have a higher catalytic efficiency than TS1 (Fig. 3d). Moreover, exogenous jasmonate treatment resulted in an obviously higher level of TS2 than TS1 transcripts (Fig. 3e), implying that TS2 could play a role in paclitaxel biosynthesis in response to different environmental and developmental cues via jasmonate signalling. Sequence identity analysis showed that TS2 shared only 77–78% protein sequence identity with TS1 and *T. brevifolia* TS (TbTS), which is much lower than the sequence similarity (over 90%) within the previously reported TS genes (Supplementary Table 21), suggesting that TS2 is a unique TS gene that diverged from TS1 and TbTS. Phylogenetic tree analysis further confirmed that a *Taxus*-specific gene duplication event approximately 33.2 Ma resulted in two distinct types of TS genes (Extended Data Fig. 5), demonstrating that TSs were encoded by two types of TS genes resulting from gene duplication events in *Taxus*. Together, these results suggest that the genes involved in the two initial steps of the paclitaxel biosynthesis pathway are arranged in a gene cluster named the ‘taxadiene gene cluster’. The taxadiene gene cluster might be formed by gene duplications and neofunctionalization in *Taxus* and may be somewhat similar to previous studies on operon-like gene clusters in plants^32,33^.

**Extended Data Fig. 6:**
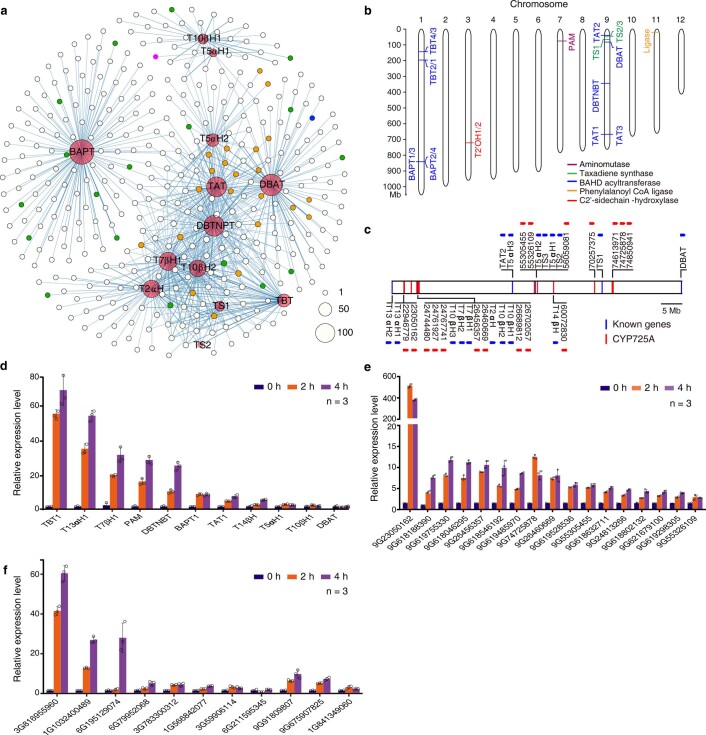
The characteristic of gene expression and location related to the paclitaxel biosynthesis in *T. chinensis* var. *mairei*. **a**, Co-expression net of paclitaxel biosynthesis genes. The genes with a Pearson correlation coefficient value above 0.75 are displayed on the net. The known paclitaxel biosynthesis genes, CYP725s, CYP450s, and the remaining genes are represented as red, orange, green, and white dots, respectively. The purple and blue dots show the two novel CYP725A genes, 55305455 and 55326109, respectively. The size of the dot correlates with the gene number. **b**, Genomic location of the annotated genes known to be involved in paclitaxel biosynthesis, except for CYP450s. The different colors of the short lines indicate the different types of annotated genes and their homologs in the paclitaxel pathway; the short purple, green, blue, orange, and red lines correspond to aminomutase, taxadiene synthase, BAHD acyltransferase, ligase, and C2’-sidechain-hydroxylase. **c**, Defined genes and 18 novel CYP725As on chromosome 9. The known genes in the paclitaxel biosynthesis pathway (known genes) are marked by blue lines, while the unknown CYP725As are shown in red lines. The arrows show gene orientations. **d-f**, The relative transcript abundance of the eleven defined paclitaxel biosynthetic genes (**d**), the sixteen CYP725A candidates (**e**), and the eight TFs and three BAHD acyltransferase genes (**f**) in MeJA-induced Taxus cell lines by quantitative real-time PCR (qPCR) analysis. The relative gene expression levels are represented as the average fold change (2^-ΔΔCt^). The Taxus actin 1 gene (7G702435613) was used as an internal reference. Error bars indicate standard errors from three independent biological replicates. Source data

Furthermore, we established a gene-to-gene coregulation network using three rounds of subtraction screening with RNA-seq datasets. The network could cover all known paclitaxel biosynthetic genes (Extended Data Fig. 6 and Supplementary Table 22), indicating its comprehensiveness and high credibility. We identified 17 CYP725A genes, 3 transferases and 10 TFs with this network, which was strongly associated with known paclitaxel biosynthetic genes (Supplementary Tables 23 and 24). Real-time quantitative PCR assays confirmed that the expression of certain genes could be induced by jasmonates (Extended Data Fig. 6), implying that their encoded proteins could be investigated as potential enzymes in paclitaxel biosynthesis. Together, these results outline the biosynthesis pathway of paclitaxel in *T. chinensis* var. *mairei* (Fig. 3f) and provide valuable genetic resources for improving paclitaxel production through genetic breeding and synthetic biology.

## Discussion

The absence of a chromosome-level genome sequence from *Taxus* has prevented in-depth phylogenomic studies of *Taxus*. Our study provides an example assembly of a complex genome in trees using various sequencing technologies on DNA from endosperm calli containing haploid chromosomes. Flow cytometry analysis indicated that the nuclear genome (2*n*) size of the diploid cells of *Taxus* was approximately 20.80–24.85 Gb, nearly twice the haploid genome size evaluated by *k*-mer analysis (Extended Data Fig. 1a). The vast majority of HiFi sequences from *Taxus* leaves (diploid) could be mapped to the haploid genome (up to 95%). Moreover, 75.81% of the 228,762,501 single nucleotide polymorphisms, 44.97% of the 847,935 insertions and deletions, and 85.64% of the 64,927 structural variants were heterozygous. Taken together with the low heterozygosity (0.02%) of *T. chinensis* var. *mairei*, these results demonstrate that the haploid genome assembly could basically represent its diploid genome, showing the advantages of using endosperm calli for genome assembly.

We found that the complete BUSCOs increased only from 64.7% to 65.2% when the N50 value was increased from 637 kilobases to 2.44 Mb during the genome assembly. The low BUSCO value might be due to the limitations of the BUSCO reference dataset. The latest dataset version is embryophyta_odb10 (10 September 2020), containing 1,614 genes from single-copy genes of 50 species, including two bryophytes (*Physcomitrella patens* and *Marchantia polymorpha*), one fern (*Selaginella moellendorffii*) and 47 seed plants (all are angiosperms) but not including any genes of gymnosperms. Consistently, across all of the reported gymnosperm genomes, except for *Gnetum montanum*, the BUSCO values were not higher than 73%, and four of these genomes had values lower than 51% (Supplementary Table 6). The BUSCO value of the *Taxus* genome was 65.2%, similar to that of *Ginkgo biloba* (69.4%) and *Pseudotsuga menziesii* (67.8%). To assess the *Taxus* genome quality more comprehensively, we mapped the Illumina DNA sequencing data (~693 Gb) for the genome survey onto the assembled genome and found that up to 99.60% of the sequencing data could be mapped, indicating the integrity of the genome assembly. Moreover, we collected transcriptome data from *Taxus* organs, comprehensively covering eight tissues and cell lines (root, stem, leaf, bark, male strobili, female strobilus, HC and LC), and mapped the sequencing data to the *Taxus* genome. The results showed that the average overall mapping rate of transcriptome data to the genome reached 90.45% (Supplementary Table 25), suggesting the integrity of functional genes in the genome.

**Extended Data Fig. 7:**
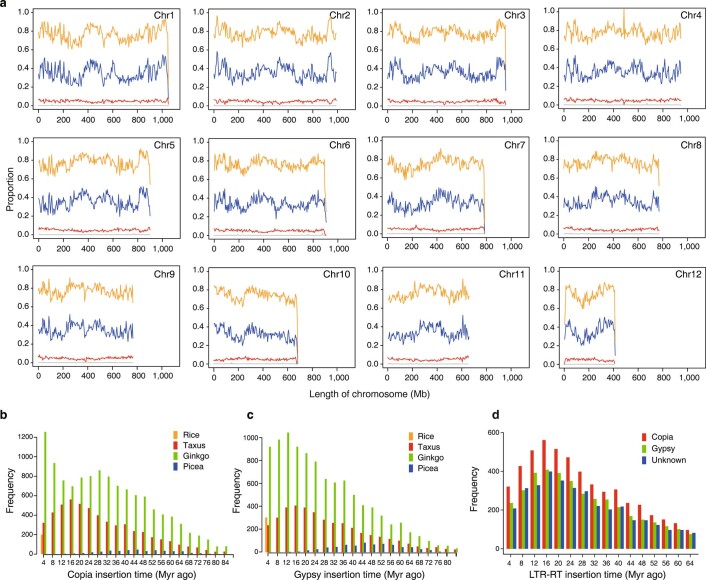
The LTR features related to Fig. 1. **a**, Distribution of repeats and LTR on the chromosomes. The lines indicate different elements (Orange: repeats; Blue: Gypsy; Red, Copia; Grey: Unknown LTR). Each point on the line represents the proportion of the component in the 5 Mb window. **b** and **c**, Comparison of distributions of LTR insertion times in different species. The histogram shows the distributions of insertion times calculated for Copia **(b)** and Gypsy **(c)** in Taxus, ginkgo, picea, and rice. The different colors of the columns represent the Copia and Gypsy insertions of the four plants. **d**, Comparison of insertion-time distributions of different LTR elements in the Taxus. The histogram shows the distributions of the insertion times calculated for the Taxus LTR elements (Gypsy, Copia, and an unknown type).

The *Taxus* genome contains 4.08 Gb of LTR retrotransposons, including 87.28% Gypsy and 12.35% Copia retrotransposons and a small proportion of unknown LTRs (0.37%) (Supplementary Table 8). The LTR distribution analysis showed that LTRs tended to be distributed throughout the entire chromosome (Extended Data Fig. 7a). In particular, Copia tends to be enriched at the two ends of the chromosomes, while Gypsy is more enriched at the chromosome ends and central areas. Compared with previous studies in groundnuts^34^, the *Taxus* genome exhibited obvious differences in LTR distribution. The LTR retrotransposons of the groundnut genome are mainly distributed in the central regions of the chromosomes, close to the centromeres. This difference may come from the large disparity in genome size and the difference between angiosperms and gymnosperms.

The LTR insertions in the *Taxus* genome mainly occurred 8 to 24 Ma during the long insertion period (4–60 Ma) (Fig.1c and Extended Data Fig. 7b–d), while the primary insertion times of LTRs in spruce and ginkgo were 12–24 and 16–24 Ma within their insertion span from 4 to 64 Ma^25,35^. These results suggest that the *Taxus* genome has a similar LTR insertion time trend to that in the spruce and ginkgo genomes. The very long insertion time phenomenon might be related to the evolutionary characteristics of gymnosperms. It is generally accepted that gymnosperms are slow-evolving plants. Their morphology is highly conserved, which is supported by the high similarity between extant species and fossil records. Previous studies have shown that angiosperms and gymnosperms differ considerably in their mutation rates of molecular evolution per unit time, with gymnosperm rates being, on average, seven times lower than those of angiosperm species^36^. For this reason, an insertion time longer than 60 million years is common in gymnosperm genomes because of the much lower mutation rate. For example, up to 8.27% of LTRs were inserted into the ginkgo genome over 60 million years, and 13.31% of LTRs were inserted into the spruce genome over 60 million years^25,35^.

In addition to CYP450 enzymes, acetyltransferases play an essential role in paclitaxel biosynthesis, especially BAHD acyltransferases. We found 127 BAHD acyltransferases by identifying their conserved motifs (HXXXD and DFGWG). The BAHD acyltransferases in *Taxus* were mainly distributed in Clades I, II, VI and V. Clade V can be divided into three groups (Groups I–III), among which Group I contains all known BAHD acyltransferases in the paclitaxel biosynthesis pathway (Supplementary Fig. 5). It would be worthwhile to investigate whether Group I contains other acyltransferases that function in paclitaxel biosynthesis in the future (Supplementary Table 26). PlantiSMASH analysis indicated that the acyltransferase genes are not organized into any gene clusters. Genomic location analysis showed that the BAHD acyltransferase genes in paclitaxel biosynthesis were mainly distributed on chromosomes 1 and 9 (Extended Data Fig. 6b). Furthermore, TAT2 was colocalized with CYP450s in gene group 9.2 (Fig. 2c and Extended Data Fig. 6b). The relationship between CYP725As and acetyltransferases in paclitaxel evolution is an interesting aspect to study in the future.

In the *Taxus* genome, a total of 34 potential gene clusters related to secondary metabolism were found, including 13 saccharides, 7 terpenes, 1 alkaloid, 1 saccharide–terpene, 1 saccharide–polyketide, 1 lignan–terpene, 1 terpene–alkaloid and 9 putative gene clusters (Supplementary Tables 20 and 27). Two gene clusters (clusters I and II) belonging to the terpene cluster were involved in paclitaxel biosynthesis because cluster I contained the TS2, TS3, T5αH2 and T5αH3 genes, and cluster II included TS1. Except for these five genes, other related enzymes in the paclitaxel synthesis pathway were not included in any gene clusters. However, we found that most of the known genes involved in paclitaxel biosynthesis, including TAT2, DBAT, TS1/2/3, T7βH1/2, T13αH1/2, T10βH1/2/3, T5αH1/2/3 and T14βH, are located on a small 71.82-Mb region on chromosome 9 (designated the T13αH2–DBAT segment: base pairs (bp) 19994572–91811351; Extended Data Fig. 6c). Therefore, many genes that play roles in different steps of the paclitaxel synthesis pathway are located in a limited genomic region, implying that there might be a coordinated regulatory mechanism of their gene expression. It would be an important future project to investigate whether the genes are organized in a larger-scale gene cluster to achieve better collaborative expression.

To date, all known TS enzymes are homologous to TS1 (amino acid homology > 90%) (Supplementary Table 21). Our study showed that TSs could be encoded by two distinct types of TS genes resulting from gene duplication events in *Taxus*. As a representative of the new type of TS enzyme, TS2 only has approximately 77–78% amino acid homology with the reported TS enzymes (Supplementary Table 21) and exhibits more robust induced expression characteristics in treatment with jasmonates (Fig. 3e). The different properties of these two types of TS enzymes imply a new *Taxus* defence regulation mechanism. In *Taxus*, the excessive synthesis of taxanes is not conducive to its growth or development, although these chemicals play an essential role in defence responses. It is therefore necessary to accurately and efficiently control the taxane level in cells in response to environmental changes. Our results provide a new hypothesis to explain the regulation of taxane levels in plant cells. When there are no biotic or abiotic stresses, jasmonate signalling is blocked, and TS1 is responsible for taxane biosynthesis to maintain taxanes at a basic level. However, once insect attack or other stresses occur, jasmonate signalling is activated, and TS2 is rapidly expressed to quickly increase the taxane content in cells.

**Extended Data Fig. 8:**
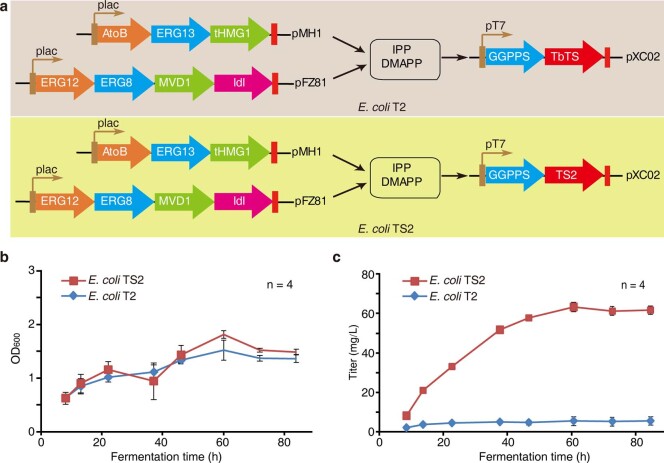
Comparison of the two types of TS on the production of taxadiene in *E. coli*. **a**, Taxadiene-producing *E. coli* T2 (harboring pMH1, pFZ81, and pXC02) was constructed by coexpressing nine genes (*AtoB*, *ERG13*, *tHMG1*, *ERG12*, *ERG8*, *MVD1*, *IdI*, *GGPPS*, and *TbTS*) in *E. coli*^29^, while *E. coli* TS2 was generated by replacing *TbTS* with *TS2* in *E. coli* T2; **b**, The cell concentrations of the strains *E. coli* T2 and TS2 were measured by OD_600_ at set intervals (at 8, 13, 22, 37, 46, 60, 72 and 84 hours); **c**, The titers of taxadiene produced by *E. coli* T2 and TS2 in shaking flasks. TbTS, a *T. brevifolia* taxadiene synthase that shares 98.42 % amino acid sequence identity with TS1, represents type I TSs, while TS2, sharing 77 % protein sequence identity with TS1, represents type II TSs. Error bars show standard error (n = 4 independent biological replicates).

In addition, we tried to explore the application potential of TS2 in bioengineering. Bian et al. reported an engineered *Escherichia* *coli* strain with TbTS (belonging to Type I) for the taxadiene product^37^. We replaced the TbTS gene with the TS2 gene (Extended Data Fig. 8a). After 60 hours of fermentation, we found that the taxadiene titre from the strain containing TS2 was over ten times higher than that from the strain containing TbTS, while the OD_600_ of the two strains was not much different (Extended Data Fig. 8b,c). This result shows the great potential of TS2 in bioengineering to produce taxadiene in the future.

We also explored the function of two unknown CYP725As (55305455 and 55326109) in the taxadiene cluster using the well-established T5αH reaction assay (Fig. 3c and Supplementary Fig. 4h). We further incubated yeast microsomes that included 55305455, T5αH1 and cytochrome P450 reductase (CPR) with taxadiene as a substrate at the same time and analysed the reaction products by gas chromatography mass spectrometry (GC–MS). As shown in Supplementary Fig. 6, we detected only 5(12)-oxa-3(11)-cyclotaxane, 5(11)-oxa-3(11)-cyclotaxane and taxa-4(20),11(12)-dien-5α-ol, which can be obtained by catalysing taxadiene by the T5αH1 enzyme. The same result was obtained with 55326109 protein in the reaction system (Supplementary Fig. 6). These results suggest that the unknown CYP725As are not involved in the subsequent reaction catalysed by T5αH. However, the tissue expression specificity of 55305455 and 55326109 was similar to that of TS and T5αH in the cluster, and both of them exhibited higher expression levels in roots than in leaf and bark tissues (Fig. 3a). The real-time PCR assay validated that their expression was induced by jasmonate in *Taxus* cells (Extended Data Fig. 6e), which is consistent with paclitaxel accumulation (Supplementary Fig. 1). Moreover, the gene-to-gene coregulation network showed that 55305455 and 55326109 were correlated with DBAT and T5αH1, respectively (Extended Data Fig. 6a). These results indicate that the two CYPs may play a role in paclitaxel biosynthesis and metabolism and are worthy of in-depth study in the future.

## Methods

### Plant materials and genome sequencing

Seeds of a single female *T. chinensis* var. *mairei* were collected from the natural range of *Taxus* (113° 89′ 55′′ N, 28° 26′ 32′′ E) in the Liuyang region, Changsha city, Hunan Province, China, in November 2015. Single embryos and endosperm were induced as calli^23,38^.

For sequencing of the haploid tissue, DNA was extracted from the endosperm callus of *T. chinensis* var. *mairei*^23^. The DNA quality was checked by agarose gel electrophoresis and a Qubit fluorimeter (Thermo Fisher). The paired-end libraries with a 500-bp insert length were prepared by following the Illumina protocols. Sequencing of the library was performed on the Illumina HiSeq 2500 system. For the PacBio Sequel analysis, SMRTbell TM libraries were prepared according to the manufacturer’s protocol for the sequencing platform. Four independent Hi-C libraries were constructed and sequenced on an Illumina HiSeq 2500 (PE125 bp) at Annoroad Gene Technology Co.

For circular consensus sequencing, genomic DNA was extracted from frozen leaves using the DNeasy Plant Mini Kit (Qiagen). A 15-kilobase DNA SMRTbell library was constructed and sequenced on a PacBio Sequel II platform; these sequencing reads are known as highly accurate long reads, or HiFi reads.

### Genome assembly and gene annotation

The uncorrected PacBio reads were assembled using wtdbg2 (ref. ^39^), the fastest sequence assembler for long noisy reads. The assembly reached the best continuity with the following parameters: -k, 0; -p, 19; -K, 5000; -S1; –aln-noskip–tidy-reads, 5000; –edge-min, 2; –rescue-low-cov-edges. The software Arrow in the GenomicConsensus package (https://github.com/PacificBiosciences/GenomicConsensus) was applied to generate the consensus sequences from the primary assembly. The raw PacBio reads were aligned to the assembly of red bean^39^ using pbalign (v.0.3.1) with the default parameters, and then the alignment was passed to Arrow (v.2.2.2) to produce the corrected assembly. The consensus process was performed iteratively twice. Further polishing of the assembly genome was conducted using Pilon^40^ with Illumina data, with the following parameters: –fix, all; –mindepth, 0.4; –K, 65; –threads, 24; –minmq, 30; –minqual, 30; –changes.

For Hi-C assembly, the clean Hi-C sequencing data were mapped to the genome draft by HiC-Pro (v.2.7.8)^41^, and the library quality was assessed by counting the number of unique valid paired-end reads. Only unique valid paired-end reads were maintained for downstream analysis. We used the Hi-C data to align and correct the contigs for misassembly through the Juicer^42^ pipeline and the 3D-DNA pipeline^43^. The assembly package Lachesis^44^ was applied to perform clustering, ordering and orienting on the basis of the normalized Hi-C interactions. For each pseudochromosome group, the exact contig order and directions were obtained through a weighted directed acyclic graph. We filled the gaps among contigs in the pseudochromosomes using TGS-Gapcloser (v.1.01)^45^ by two rounds with continuous long-read and HiFi data (26 Gb), respectively. After the filling progress, we further removed the redundant contigs that were not anchored to the chromosomes using Purge Haplotigs (v.1.03)^46^.

For assembly assessment, the RNA-seq reads of eight tissues (including female strobilus, female leaf, female bark of stem, female root, male strobili, male leaf, male bark of stem and male root) and HC and LC were mapped to assess the assembly quality. The average mapping rate of all RNA-seq datasets was subsequently calculated by software HISAT2 (ref. ^47^) with the following parameter: score-min, L, 0, −0.1.

For repeat annotation and analyses, repetitive elements in the *Taxus* genome were identified through a combination of de novo and homology-based approaches. De novo prediction of repeat elements was carried out using RepeatModeler (v.1.0.1, http://www.repeatmasker.org/RepeatModeler/). For homology-based annotation, the repeat element libraries from Repbase^48^, the Institute for Genomic Research^49^ and the annotated *Ginkgo biloba* genome were merged with the de-novo-derived library to create the whole dataset. The dataset was then used to mask identified TEs in the *Taxus* genome with RepeatMasker (v.4.0.5, http://www.repeatmasker.org). We identified LTRs with the LTR_retriever method^50^. Specifically, LTR_finder^50^ and LTRharvest^51^ were first used to identify all the existing LTR sequences in the *Taxus* genome according to the basic sequence rules of LTRs. The candidate LTR RTs were filtered to remove non-LTR RT repeat elements or those with large amounts of tandem repeats or gaps. Especially in fragmented genome assemblies, these requirements hugely reduce the number of LTR RT candidates but ensure that only full-length LTR RTs are analysed. We integrated the results and discarded false positives using the LTR_retriever pipeline; we then estimated insertion times (*T*) on the basis of *T* = *D*/2*μ*, where *D* is the divergence rate and *μ* is the neutral mutation rate (7.34573 × 10^−10^)^36^.

For the annotation of protein-coding genes, gene structure prediction was performed using ab initio, homology-based and RNA-seq-based pipelines. For the ab initio annotation, SNAP^52^, Augustus^53^ and GlimmerHMM were applied. Eight species (*Arabidopsis thaliana*^54^, *Oryza sativa*^55^, *Gnetum montanum*^56^, *Picea abies*^25^, *Ginkgo biloba*^35^, *Selaginella moellendorffii*^57^, *Pinus taeda*^58^ and *Amborella trichopoda*^59^) were chosen for homology annotation to predict protein-coding genes using GeneWise^60^. To generate annotation results based on transcripts, RNA-seq alignment files were generated using TopHat2 (ref. ^61^) and assembled via Cufflinks^62^, and the program PASA^63^ was used to align spliced transcripts and annotate candidate genes. Finally, gene models predicted from three approaches were merged by EVM^64^. The functions of protein-coding genes were identified by mapping sequences against the Gene Ontology^65^, InterProScan^66^, Swiss-Prot (http://www.uniprot.org/)^67^, TrEMBL^68^ and TAIR databases^69^.

### Identification of WGD

Genome-wide duplications were searched in the *Taxus* genome. Self-alignment of the assembled genome sequence was performed using metablast as described previously^70^. All-versus-all paralogue analysis in the *Taxus* genome was performed using reciprocal best hits from primary protein sequences by self-Blastp in *Taxus*. Reciprocal best hits are defined as reciprocal best Blastp matches with an *E*-value threshold of 10^−5^, a *c*-score (Blast score/best Blast score) threshold of 0.3 (ref. ^71^) and an alignment length threshold of 100 amid acids. The value of *K*_s_ of reciprocal best hit gene pairs was calculated on the basis of the YN model in *KaKs*_Calculator v.2.0 (ref. ^72^). Synteny analysis was performed on *Taxus* protein-coding genes using MCScanX^73^ to identify WGD events with the default parameters from the top ten self-Blastp hits. *K*_s_ and 4DTv were calculated for *Taxus* syntenic block gene pairs.

### Genome mining for CYP450s and gene clusters

For the identification and classification of CYP450 genes, hmmsearch was used to identify CYP450 genes in the *Taxus* genome with PF00067 from the Pfam database^74^. The classification of the 649 CYP450 genes was executed by alignment with the CYP450 database^75^ using standard sequence similarity cut-offs, with definite standards of 97%, 55% and 40% for allelic, subfamily and family variants, respectively. According to the standardized CYP450 nomenclature^76^, CYP450s were divided into A-type and non-A-type CYP450s, and phylogenetic analysis of CYP450 genes was performed for A-type and non-A-type CYP450s. Neighbour-joining phylogenetic trees were constructed using the MEGA7 package with homologous amino acid sequences^77^.

For genome mining for gene clusters involved in plant specialized metabolism, PlantiSMASH^31^ was used to search for potential gene clusters using the default parameters and the GFF (General Feature Format) annotation files of the software. Gene groups were identified by in silico analysis on the basis of the following criteria: (1) the distance between two adjacent CYP450 genes in one group should be less than 5.26 Mb, and (2) one group should contain at least seven CYP450 genes.

### RNA-seq data analysis for candidate genes in the paclitaxel biosynthesis pathway

All tissues, including female strobilus, leaf, bark of stem, and root and male strobili, leaf, bark of stem, and root, were mapped to the *Taxus* genome, and the fragments per kilobase of transcript per million mapped reads value was calculated using HISAT2 and StringTie^76^. Expression data from female bark, female roots and female leaves were used to identify the genes associated with paclitaxel biosynthesis. First, we selected genes that were more highly expressed in roots or bark than in leaves. Second, the genes were further confirmed in two *Taxus* half-sib cell lines (HC and LC) with distinct accumulation patterns of paclitaxel, and the genes should be highly expressed in HC. The differentially expressed genes were filtered using edgeR^77^ with logFC > 1 and FDR < 0.05. We obtained 1,638 genes that met the above thresholds. Gene-to-gene networks were constructed using the expression matrix from MeJA-induced cell line (0, 2, 4, 8 and 24 h) RNA-seq data. Pearce correlation analysis was performed with the known functional genes as the target genes. Hypothesis development for the Pearson correlation was performed, and pairs with P < 0.05 remained.

### Functional characterization of TS genes

The open reading frames of *TS1* (*ctg6088_gene.1*) and *TS2* (*ctg5306_gene.4*) were cloned by PCR with reverse transcription from the *Taxus* cell line. Plant-Ploc (http://www.csbio.sjtu.edu.cn/bioinf/plant-multi/), ChloroP (http://www.cbs.dtu.dk/services/ChloroP/) and TargetP (http://www.cbs.dtu.dk/services/TargetP/) were used for the prediction of the plastidial target sequence. The 60-residue N-terminally truncated *TS1* and *TS2* genes were inserted into the *E. coli* expression vector pET28b to form the constructs pET28b::*TS1* and pET28b::*TS2*, respectively. All expression plasmids were constructed using the Hieff Clone One Step Cloning Kit (YEASEN), and the primers used in this work are given in Supplementary Table 28. For the in vitro enzyme assay, the enzyme assays were performed in a final volume of 500 µl of buffer (25 mM HEPES, pH 8.5, 10% glycerol, 5 mM DTT, 5 mM sodium ascorbate, 5 mM sodium metabisulfite and 1 mM MgCl_2_) containing 100 µg of purified protein and 100 µM GGPP (Sigma-Aldrich). The reaction mixture was overlaid with 500 µl of pentane (Macklin, GC–MS grade) and incubated overnight at 32 °C. In addition, the mixture was vortexed, and the pentane overlay was subsequently removed by centrifugation at 5,000 r.p.m. for 10 min and concentrated by N_2_ gas before GC–MS analysis. Inactivated TSs-His6 was used as the control. Taxa-4(5),11(12)-diene (**1**) and taxa-4(20),11(12)-diene (**2**) preparations were performed according to a previous study with the taxadiene-producing *E. coli* strain T2 (harbouring pMH1, pFZ81 and pXC02)^37^. The organic solutions containing crude compounds **1** and **2** were concentrated on ice under N_2_ gas and redissolved in dimethyl sulfoxide for the purification of compounds **1** and **2** by thin layer chromatography. The purity and concentration were determined by GC–MS.

For the determination of kinetic parameters, standard enzyme assays were carried out in a total volume of 100 μl containing buffer (25 mM HEPES, pH 8.5, 10% glycerol, 5 mM DTT, 5 mM sodium ascorbate, 5 mM sodium metabisulfite and 1 mM MgCl_2_), 36 µg (TS1) or 17 µg (TS2) of recombinant proteins and seven different concentrations of GGPP (0.2, 0.5, 1, 2.5, 5, 10, 25 and 50 µM), which were spiked with [^1-3^H]-GGPP (American Radiolabeled Chemicals, 30 Ci mM^−1^). Hot [^1-3^H]-GGPP was diluted 400 times using cool GGPP (Sigma, 1 mg ml^−1^). The reaction mixtures were incubated at 32 °C for 30 min and then quenched for 10 min using 100 µl of stop solution (containing 1 M EDTA and 4 M NaOH). The reaction mixture was extracted with 800 µl of *n*-hexane (vortexed for 10 s at 12,000 r.p.m. for 2 min), and 400 µl of the *n*-hexane layer was subsequently removed and mixed with 2 ml of the liquid scintillation cocktail. The total radioactivity of the reaction products was measured using a liquid scintillation counter (Tri-Carb 2910TR, Perkin Elmer). The kinetic constant was calculated by a nonlinear regression fit to the Michaelis–Menten equation using OriginPro v.8.6 (OriginLab)^78^.

*E. coli* TS2 was constructed by replacing *TbTS* with *TS2* on the basis of the previous taxadiene-producing *E. coli* TbTS (harbouring pMH1, pFZ81 and pXC02 and coexpressing nine genes—*AtoB*, *ERG13*, *tHMG1*, *ERG12*, *ERG8*, *MVD1*, *IdI*, *GGPPS* and *TbTS*—in *E. coli*)^37^ (Extended Data Fig. 8a). The *E. coli* strains T2 and TS2 were cultivated in 50-ml flasks containing 30 ml of LB medium at 37 °C with 100 mg l^−1^ ampicillin, 50 mg l^−1^ kanamycin and 34 mg l^−1^ chloramphenicol. When the OD_600_ reached approximately 0.1, 1 mM isopropyl β-d-1-thiogalactopyranoside was added to the cultures along with 3 ml of dodecane; the bacteria were then cultivated at 28 °C. The experiments were repeated four times. For cell concentration (OD_600_) and taxadiene measurement, 100-μl cultures and 30-μl organic layers were collected at set intervals (at 8, 13, 22, 37, 46, 60, 72 and 84 h). The produced taxadiene was detected with GC–MS and quantified with the nonyl acetate standard (Aladdin).

### Reporting Summary

Further information on research design is available in the Nature Research Reporting Summary linked to this article.

## Supplementary information

Supplementary Information Supplementary Methods, Figs. 1–6, Tables 1–4, 6–8 and 27, and references.

Reporting Summary

Supplementary Tables Supplementary Tables 5, 9–26 and 28.

Supplementary Data 1 Unprocessed western blots for Supplementary Fig. 4. These images were collected from the TANON 5200 Automatic Chemiluminescence Imaging Analysis System.

## Data Availability

The *T. chinensis* var. *mairei* genome project has been deposited in the Genome Sequence Archive at the National Genomics Data Center, and is accessible at http://bigd.big.ac.cn/ under BioProject no. PRJCA003841. Whole-genome and RNA-seq data were deposited in the Genome Sequence Archive database under accession nos CRA004292, CRA003496 and CRA004255. The *T. chinensis* var. *mairei* genome data have also been deposited at NCBI under BioProject no. PRJNA730337 and are publicly accessible at https://www.ncbi.nlm.nih.gov/Bioproject/?term=PRJNA730337. Source data are provided with this paper.

## Source data

Source Data Fig. 3 Statistical source data.
Source Data Extended Data Fig. 6 Statistical source data.

## Code availability

In-house Python and R scripts for gene location, P450 analysis and heat-map analyses can be freely downloaded at GitHub (https://github.com/liaoqinggang/Taxus_genome_pipelines).
