# Supplementary material for: The Taxus genome provides insights into paclitaxel biosynthesis
Source: Nat Plants. 2021 Jul 15;7(8):1026–36. doi: 10.1038/s41477-021-00963-5 (PMC8367818; doi:10.1038/s41477-021-00963-5)
Supplement: Supplementary file 1 — Supplementary Methods, Figs. 1–6, Tables 1–4, 6–8 and 27, and references. [file 41477_2021_963_MOESM1_ESM.pdf]

nature portfolio

https://doi.org/10.1038/s41477-021-00963-5

Supplementary information

The *Taxus* genome provides insights into paclitaxel biosynthesis

In the format provided by the authors and unedited

Supplementary information

The *Taxus* genome provides insights into paclitaxel biosynthesis

Xingyao Xiong[1,2,9], Junbo Gou[2,9], Qinggang Liao[2,9], Yanlin Li[1,3,9], Qian Zhou[2,4], Guiqi Bi[2], Chong Li[2], Ran Du[2], Xiaotong Wang[2], Tianshu Sun[2], Lvjun Guo[5], Haifei Liang[2], Pengjun Lu[2], Yaoyao Wu[2], Zhonghua Zhang[6], Dae-Kyun Ro[2,7], Yi Shang[8], Sanwen Huang[2*], Jianbin Yan[2*]

[1]College of Horticulture, Hunan Agricultural University, Changsha 410128, China.

[2]Shenzhen Branch, Guangdong Laboratory for Lingnan Modern Agriculture, Shenzhen Key Laboratory of Agricultural Synthetic Biology, Genome Analysis Laboratory of the Ministry of Agriculture and Rural Affairs, Agricultural Genomics Institute at Shenzhen, Chinese Academy of Agricultural Sciences, Shenzhen 518124, China.

[3]Engineering Research Center for Horticultural Crop Germplasm Creation and New Variety Breeding, Ministry of Education, Changsha 410128, China.

[4]Peng Cheng Laboratory Artificial Intelligence Research Center No.2, Xingke 1st Street, Nanshan, Shenzhen, 518055, China.

[5]MOE Key Laboratory of Bioinformatics, Tsinghua-Peking Joint Center for Life Sciences, School of Life Sciences, Tsinghua University, Beijing 100084, China.

[6]College of Horticulture, Qingdao Agricultural University, Qingdao 266109, China.

[7]Department of Biological Sciences, University of Calgary, Calgary, AB, T2N1N4, Canada.

[8]The AGISCAAS-YNNU Joint Academy of Potato Sciences, Yunnan Normal University, Kunming, China.

[9]These authors contributed equally to this work.

[*]Correspondence to: jianbinlab@caas.cn (J.Y.); huangsanwen@caas.cn (S.H.)

This PDF file includes:

1. Supplementary Methods
2. Additional Supplementary Figures 1, 2, 3, 4, 5, 6
3. Supplementary Tables 1-4, 6-8, 27
4. Supplementary References 1-38

1. Supplementary Methods

RNA sequencing (RNA-seq) for genome structure annotation and expression analysis

Samples of *T. chinensis* var. *mairei* from one male plant and one female plant collected in Hunan Province (GPS: N 113° 89′ 55″, E 28° 26′ 32″) were used for transcriptome sequencing. Eight tissues (female strobilus (mfs), leaf (mfl), bark of stem (mfb), and root (mfr) from the female plant, and male strobili (mtf), leaf (mtl), bark of stem (mtb), and root (mtr) from the male plant) were collected individually. Two half-sib cell lines (HC, LC) were induced from the embryo of the female plant mentioned above; the taxoid contents of the lines were different from each other[1]. The total RNA of these materials was isolated independently using TRIzol® reagent (Invitrogen). The integrity and purity of total RNA were assessed using horizontal agarose electrophoresis and an Agilent Bioanalyzer 2100 (Agilent Technologies, INC). The NEB Next UlturaTM RAN Library Prep Kit (NEB, USA) was used to construct the sequencing library. All RNA-seq libraries were sequenced using the Illumina 2500 platform. The details of the RNA-seq data of each sample are given in Supplementary Table 25.

Genome survey by *k*-mer analysis

Analysis of *k*-mer frequency was adopted to estimate the genome size by using WGS Illumina reads. The *k*-mer frequency was calculated by Jellyfish (v 2.0.0)[2], and the genome size was estimated based on the formula (Total *k*-mer counts)/(Peak depth).

Long-read variant calling for genome assessment

The HiFi reads were advantageous for variant calling because they can span repetitive or other problematic regions. HiFi reads of the Taxus leaves (diploid) were aligned to the Taxus assembly genome to assess the quality. Calling the variants were used to check the coverage of the genome, we aligned HiFi sequencing reads with Minimap2[3] and called SNP (single nucleotide polymorphism) and InDel with bcftools (v1.9)[4]. Then, the SVs (structure variants) were called using Sniffles (v1.0.12) (https://github.com/petabi/sniffles).

Analysis of the insertion time of LTR elements

The candidate LTR-RTs were identified using LTR_Finder (v1.02)[5] (parameters: -D 2000 -d 1000 -L 3500 -l 100 -p 20 -C -M 0.8) and LTRharvest[6] (parameters: -similar 90 -vic 10 -seed 20 -seqids yes -minlenltr 100 -maxlenltr 7000 -mintsd 4 -maxtsd 6 -motif TGCA -motifmis 1). Only full-length LTR-RTs were retained. In addition, false positives were discarded by the LTR_retriver pipeline[7]. After removing non-LTR repeat elements and tandem repeats, the full-length LTR retrotransposons with optimal frames were translated into amino acids. Their functional domains were predicted using HMMER[8] based on the Pfam database. Paralogs of the RT domain, specific for the Copia and Gypsy superfamilies, were then detected by the functional domain orders.

The RT protein sequences were aligned using MUSCLE (v3.8.31)[9], and maximum likelihood trees were built using FastTree[10] for the Copia and Gypsy superfamilies. The two ends of these LTR retrotransposons were aligned using MUSCLE[5,6], and the nucleotide distance (D) was estimated using the Kimura two-parameter (K2p) criterion[11], which was implemented in the distmat program of the EMBOSS package (v6.6.0)[12]. The rates of nucleotide substitution (μ) were obtained from a previous study[13]. The insertion times of an LTR retrotransposon were calculated using $T = D/2\mu$.

Gene expression level analysis

RNA-seq reads were aligned to the Taxus genome using HISAT2 (v2.1.0)[14] with default parameters. The sequence alignment files generated by HISAT2 were subsequently inputted to StringTie (v1.3.5)[15] to generate counts of uniquely mapped reads of annotated genes from the reference annotation file. The genes that differed significantly between two tissues were identified with count-based methods in R packages edgeR (v3.18.1)[16]. Benjamini-Hochberg correction was used to correct for multiple comparisons (with a false discovery cut-off < 0.05). The read counts of genes were transformed to FPKM values using fragments mapping per kilobase of exon per million fragments mapped. The FPKM values were used to represents the expression level of genes. Whereas, the genes with high sequence similarity were difficult to separate the reads mapping on these genes based on the methods above. To detected the high similarity sequence genes, we adjusted the threshold (the min acceptable alignment score parameter --score-min set as L,0, 0 in HISAT2) to no mismatch and get the alignment files. The

expression level of genes was calculated using StringTie (v1.3.5)[15] using the alignment file as input files. Then, the relative low nucleotide differences distributed on the exons could use to distinguish the different expression level

Ortholog analysis

Publicly available genome and annotated peptide sequences of *A. thaliana* (TAIR 10), *O. sativa* (IRGSP-1.0), *A. trichopoda* (AMTR1.0), and *S. moellendorffii* (v1.0) were downloaded from EnsemblPlants (http://plants.ensembl.org/), while the *P. abies* (v1.0b) and *G. biloba* (v1.0) sequences were downloaded from TreeGenes[17]. An all-to-all blast was performed using blastall[18] and the data were then processed to find orthologous gene groups using OrthoMCL[19]. Subsequently, 193 single-copy gene families (out of a total of 35,298 gene families) were found, and their protein sequences were aligned with MUSCLE[20]. The aligned sequences were then integrated into a single concatenated sequence for each species, and the conserved blocks were identified for further phylogenetic analysis using Gblocks[21]. The maximum likelihood phylogenetic tree was constructed using RAxML (Random Accelerated Maximum Likelihood)[22] with the GAMMAI+LG model, and the absolute rates of divergence times were estimated using r8s[23]. The number of expanded or contracted gene families along each branch was computed using CAFÉ[24] with a *p*-value cutoff of 0.05. Gene Ontology enrichment analysis was performed and visualized using clusterProfiler[25].

Expression and purification of TSs in *E. coli*

To obtain recombinant TSs-His6, pET28b::*TS1* and pET28b::*TS2* were transformed into *E. coli* BL21 (DE3) and streaked on LB plates with kanamycin (50 μg mL^{-1}). A single positive colony was cultured at 37°C overnight in 5 mL of LB liquid medium with the same kanamycin concentration. The overnight cultures were used for expanding the culture in 500 mL of fresh medium and grown until the OD_{600} reached 0.4, at which point, 1 mM isopropyl *β*-D-1-thiogalactopyranoside (IPTG) was added to induce expression. Expression induction was performed in a 180 rpm shaker at 16°C overnight, and harvested cells were resuspended in lysis buffer (50 mM sodium phosphate, pH 8.0 and 300 mM NaCl and 10 mM imidazole). After cell lysis by sonication and centrifugation at 4,000 g for 20 min at 4°C, the total protein supernatant

was loaded onto a 1.5 mL HisPur Ni-NTA resin-packed column (Thermo Scientific, U.S.A), and the recombinant TSs-His6 were finally eluted with elution buffer (50 mM sodium phosphate, pH 8.0, 300 mM NaCl and 250 mM imidazole). The purified recombinant TSs-His6 were desalted into enzyme assay buffer (25 mM HEPES, pH 8.5, 10% glycerol, 5 mM DTT, 5 mM sodium ascorbate, 5 mM sodium metabisulfite, and 1 mM $MgCl_2$) and concentrated by centrifugation with a 30-kDa cutoff Millipore concentrator. The content and purity of the recombinant TSs-His6 were determined by a BCA protein assay kit (Beyotime, China).

***In vitro* characterization of T5αH1 and its homologous genes**

The ORFs of Taxus *T5αH1* (*ctg5306_gene.3*), *T5αH2* (*ctg7747_gene.2*), *T5αH3* (*ctg2768_gene.2*), *55326109* (*ctg7747_gene.3*), and *55305455* (*ctg7747_gene.4*) were cloned from the Taxus cell line cDNA and inserted into the yeast expression vector pESC-His. Thus, the recombination plasmid pESC-His-*CYP725As-Flag* was obtained after positive clone screening. pESC-His-*CYP725As-Flag* was expressed in the yeast WAT11 strain[26]. Their expression was confirmed by western blot assay. All expression plasmids were constructed using the Hieff Clone™ One Step Cloning Kit (YEASEN, China) and primers used in this work are given in Supplementary Table 28.

For the *in vitro* enzyme assay, microsomes of the yeast strain WAT11 expressing pESC-His-CYP725As-Flag were prepared as described previously[27]. As a control, microsomes of the WAT11 strain harboring the empty vector pESC-His were prepared. The activity of the pESC-His-CYP725As-Flag protein was tested in a 1 mL mixture containing 50 mM HEPES buffer (pH 7.5), 100 μM taxadiene substrate, 500 μM NADPH, and 2 mg of pESC-His-CYP725As-Flag-containing microsomal proteins and overlaid with 500 μL of pentane. As a control, microsomes harboring the empty vector pESC-His were prepared. After incubating the mixture at 32°C for 2 h, the pentane layer was separated for GC/MS analysis.

The previous peroxide and stability assays indicated that OCT could be oxidized from taxa-4(5),11(12)-diene by hydrogen peroxide, and OCT can spontaneously form iso-OCT at 37°C overnight[28]. For given OCT and iso-OCT, 100 μL of the above purified taxadienes (approximately 200 ug L^{-1}) were concentrated by N_2 gas in a glass GC vial, then the residue was

resuspended in 500 μL of hydrogen peroxide and shaken at 37°C and 220 rpm overnight. After reacting overnight, the taxanes were extracted with hexane and analyzed with GC-MS.

Transcription analysis under methyl jasmonate (MeJA) treatment

The *T. chinensis* var. *mairei* LC cell lines were divided into two groups: the test group was treated with 100 μM MeJA solution, whereas the control group was treated with solvent (ethanol). Samples were harvested after 0, 2, 4, 8, and 24 h of elicitation for gene expression analysis and taxane measurements. Experiments were carried out in triplicate. Total RNA was isolated from the MeJA-treated samples using the EASYspin Plant RNA isolation kit (Aidlab, Beijing, China), and 1 μg of cDNA was synthesized using Hifair® III 1st Strand cDNA Synthesis SuperMix for qPCR (gDNA digester plus) (YEASEN, China). Transcript abundance was measured using a QuantStudio™ 3 System with Hieff® qPCR SYBR® Green Master Mix (Low Rox Plus) (YEASEN, China). All real-time PCRs were repeated with at least two technical and three biological replicates. Mean cycle threshold (*Ct*) values were normalized using *T. chinensis* var. *mairei* actin 1 gene (*7G702435613*) as a validated reference gene. The relative gene expression value was calculated using the $2^{-\Delta\Delta Ct}$ method. Gene-specific primers are listed in Supplementary Table 28.

Metabolite extraction and chromatography-mass spectrometry analysis

The cell samples were extracted with a modified Wolfender method. One hundred milligrams of freeze-dried cell powder was weighed and transferred into a 2-mL centrifuge tube with 10 μL internal standards (500 ng mL^{-1} dexamethasone). Then, the samples were resuspended in 1.5 mL extraction buffer (methanol:water = 80:20, v/v), and a 30 min ultrasonic-assisted extraction process was applied. After 15 min of centrifugation at 14,000 g, the supernatants were dried in a LABCONCO CentriVap vacuum centrifugal concentrator and resuspended in 200 μL methanol solvent (80:20, v/v).

GC/MS analysis was performed on an Agilent 7890B GC machine (Agilent Technologies, Waldbronn, USA) equipped with an Agilent 7000C mass selective detector at 70 eV and 1.2 mL min^{-1} helium flow. One to five microliters of the sample was injected and analyzed on an Agilent HP-5MS column (5% phenyl methyl silox, 30 m × 250 μm internal diameter, 0.25-μm film

thickness). The oven temperature program was as follows: 45°C for 1 min, then a 10°C min^{-1} ramp to 250°C, and a hold at 250°C for 5 min. The injection temperature was 250°C. Full mass spectra were generated for metabolite identification by scanning the mass-to-charge ratio (*m/z*) range from 40 to 350.

Liquid chromatography-mass spectrometry (LC-MS) analysis was carried out on an ACQUITY UPLC I-Class (Waters) coupled to a 4500 QTRAP triple quadrupole mass spectrometer (AB SCIEX) equipped with a 50 × 2.1 mm, 1.8 µm ACQUITY UPLC™ Waters Xselect HSS T3 column (Waters). Ten microliters of supernatant was loaded each time and then eluted at a flow rate of 200 µL min^{-1} with initial conditions of 40% mobile phase A (0.1% formic acid in methanol) and 60% mobile phase B (0.1% formic acid in water) followed by a 10-min linear gradient to 100% mobile phase B. The autosampler was set at 10°C. Mass spectrometry was operated separately in positive electrospray ionization mode. The analyte [M+H] was selected as the precursor ion. The quantitation mode was multiple reaction monitoring (MRM) mode using mass transitions (precursor ions/product ions). The temperature of the ESI ion source was set at 500°C. The curtain gas flow was set at 20 psi, collisional activated dissociation (CAD) gas was set as the medium, and the ion spray voltage was (+) 5,500 V, with ion gases 1 and 2 set as 50 psi. AB SCIEX Analyst 1.6.3 Software (Applied Biosystems) was used for data acquisition and processing.

2. **Additional Supplementary Figures**

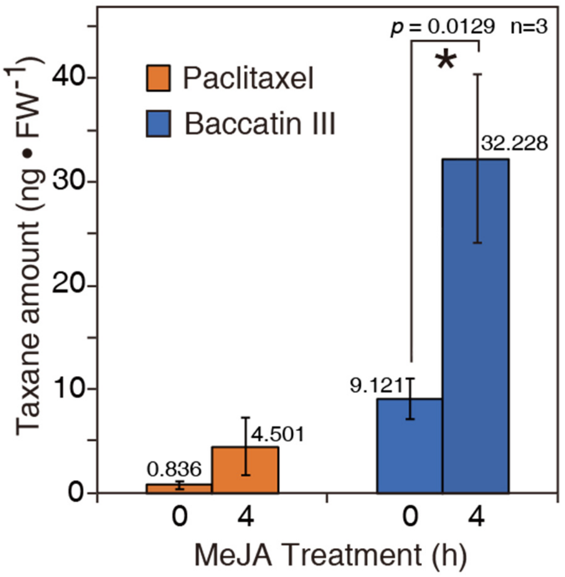

Additional Supplementary Fig.1 The accumulation of paclitaxel and baccatin III in Taxus cell line induced by 100 μM MeJA at 0 hours and 4 hours. The amounts of paclitaxel and baccatin III were measured by LC-MS analysis. FW indicates fresh weight (100 mg); error bars display the standard error (n = 3 biological replicates); asterisks show significant differences with $*p \leq 0.05$ by two-tailed Student's t-test.

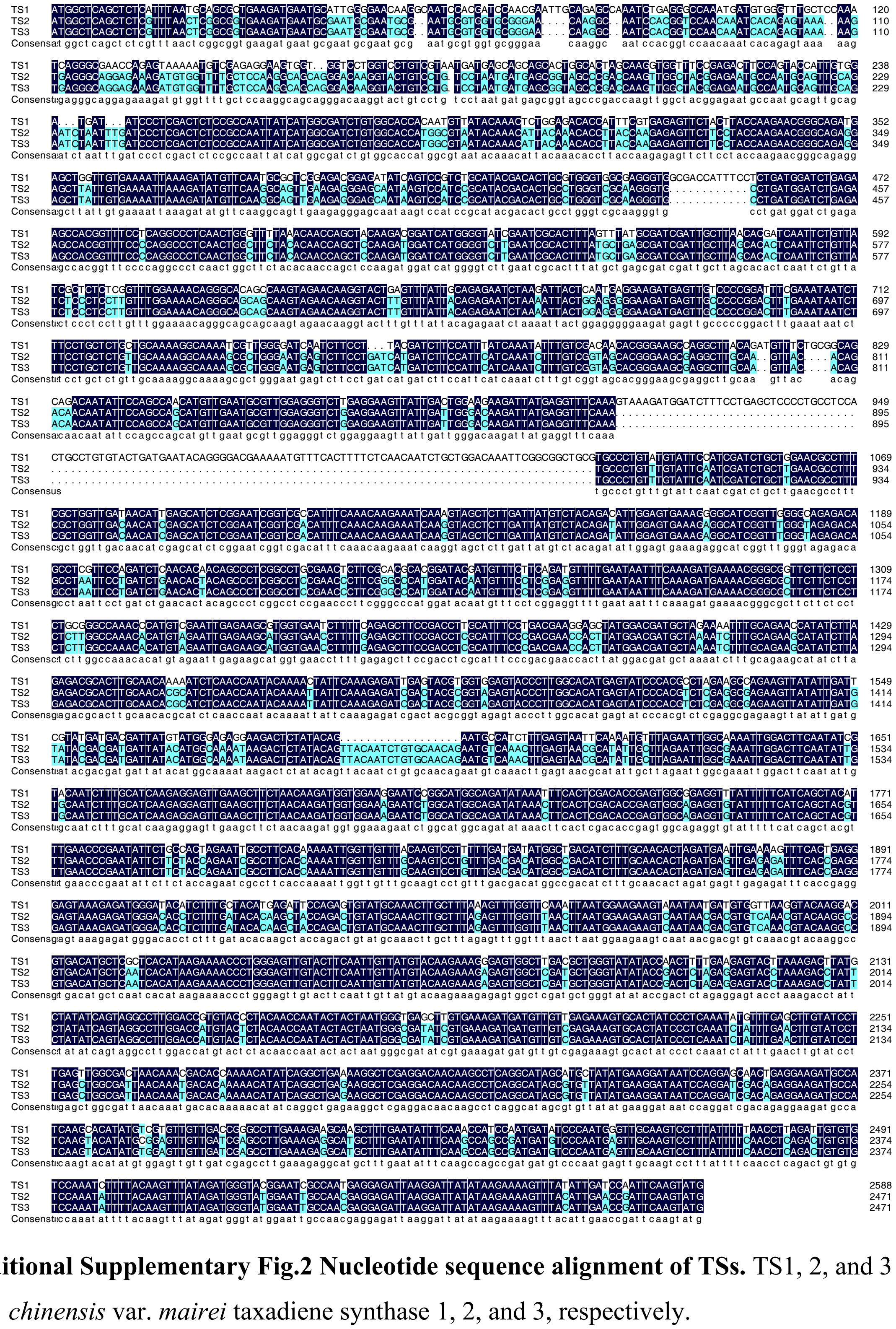

Additional Supplementary Fig.2 Nucleotide sequence alignment of TSs. TS1, 2, and 3 refer to *T. chinensis* var. *mairei* taxadiene synthase 1, 2, and 3, respectively.

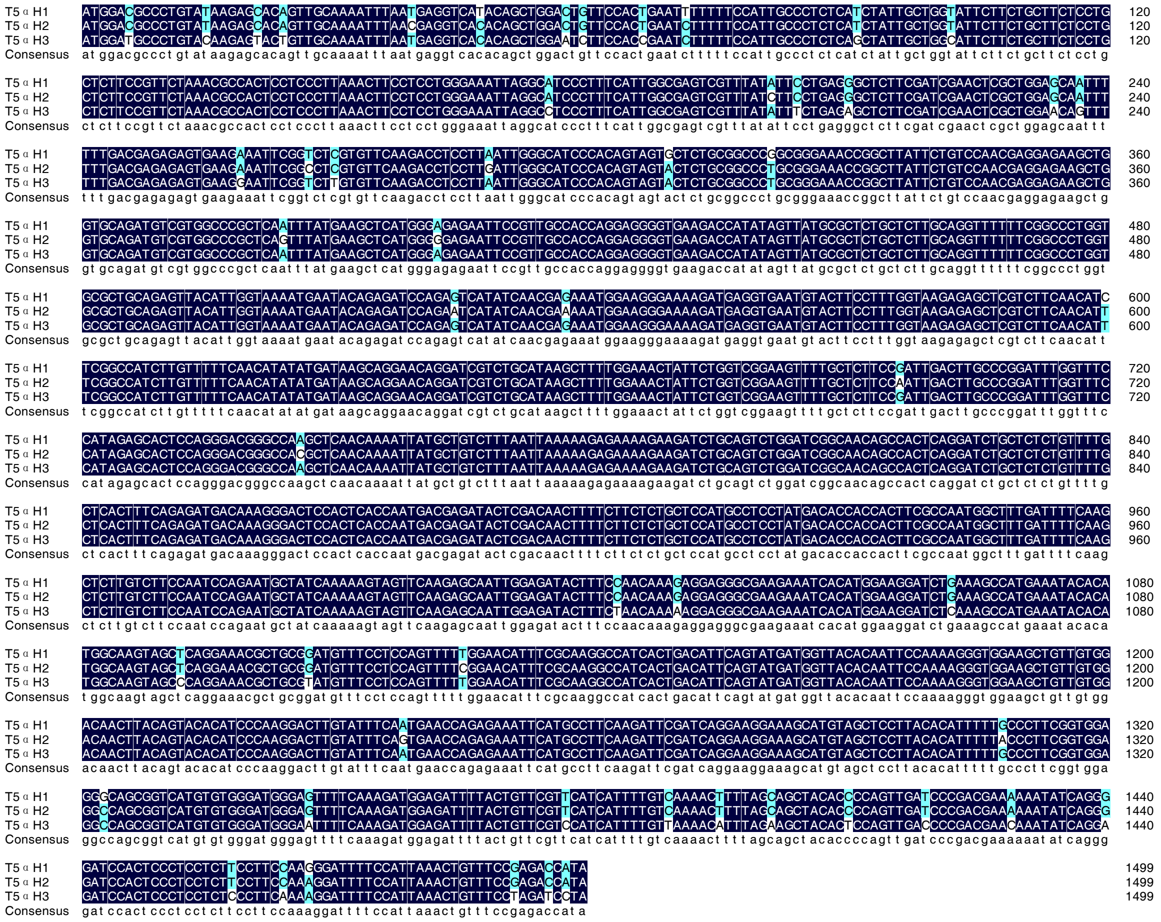

Additional Supplementary Fig.3 Nucleotide sequence alignment of T5αHs. T5αH1, 2, and 3 refer to *T. chinensis* var. *mairei* taxa-4(5),11(12)-diene-5α-hydroxylase 1, 2, and 3, respectively.

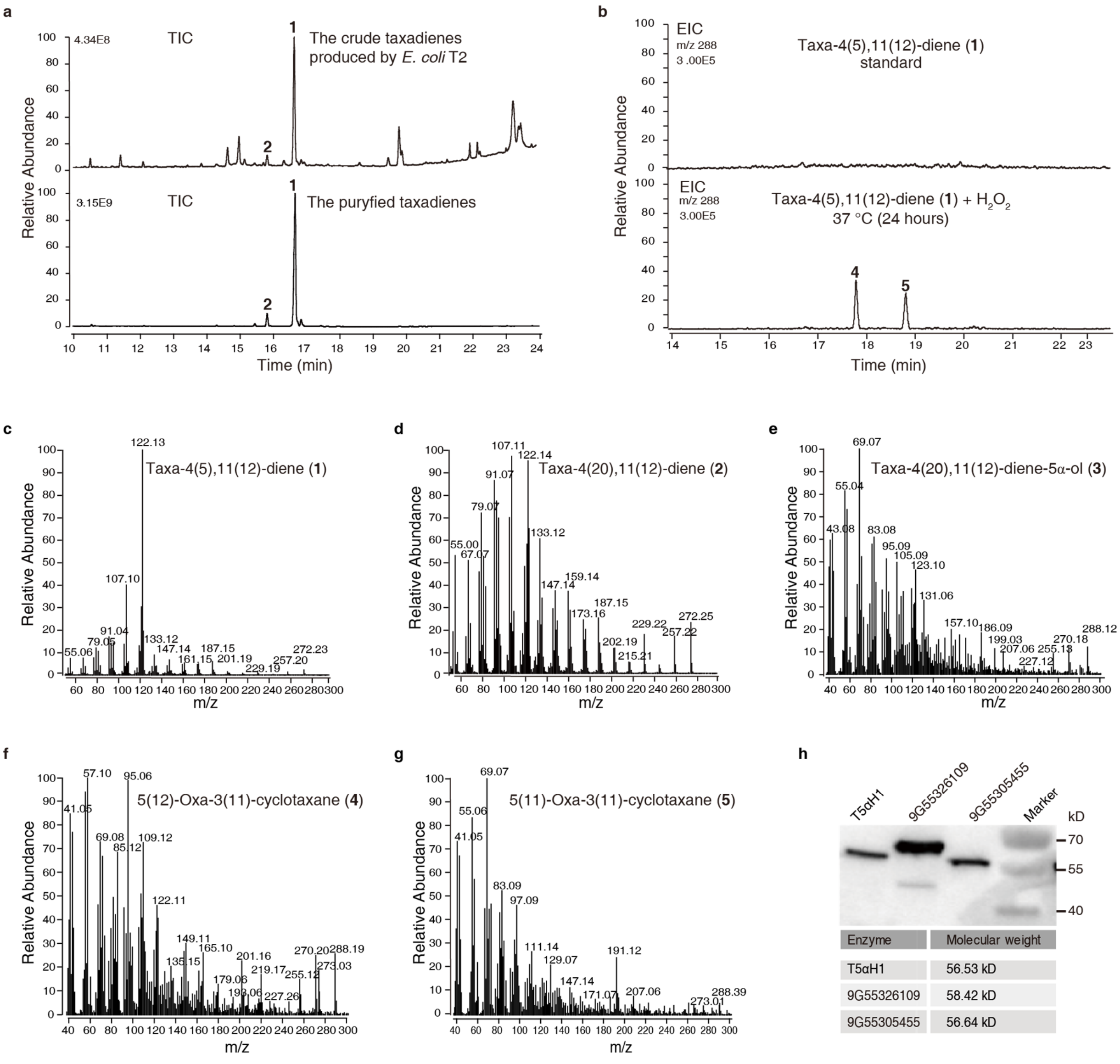

Additional Supplementary Fig.4 GC-MS analysis of taxanes (peaks 1, 2, 3, 4, and 5) and protein expression of the three CYP725As (T5αH1, 9G55326109, and 9G55305455) in yeast to complement Fig. 3. a, GC-MS analysis of the crude taxadiene produced by *E. coli* T2[25] and the purified taxadiene by thin-layer chromatography. Peaks **1** and **2** are taxa-4(5),11(12)-diene and taxa-4(20),11(12)-diene, respectively, produced by TSs; **b**, GC-MS analysis of preparative **4** and **5**. OCT (**4**) was oxidized from **1** by hydrogen peroxide, and **4** spontaneously formed iso-OCT (**5**) at 37°C overnight. Peaks **3**, **4** and **5** represent the compounds taxa-4(20),11(12)-diene-5α-ol, OCT and iso-OCT, respectively, generated by T5αHs. Chromatograms at the extracted ions of m/z^+ 288. The MS/MS fragmentation of peaks **1** (**c**), **2** (**d**), **3** (**e**), **4** (**f**) and **5** (**g**). **h**,

Immunoblotting assays of three enzymes (T5αH1, 9G55326109, and 9G55305455) in the WAT11 strain. The mouse monoclonal antibody for the FLAG tag and sheep polyclonal antibody conjugated with horseradish peroxidase (Beyotime, China) were used as primary and secondary antibodies, respectively. The immunoblotting assays were repeated 3 times.

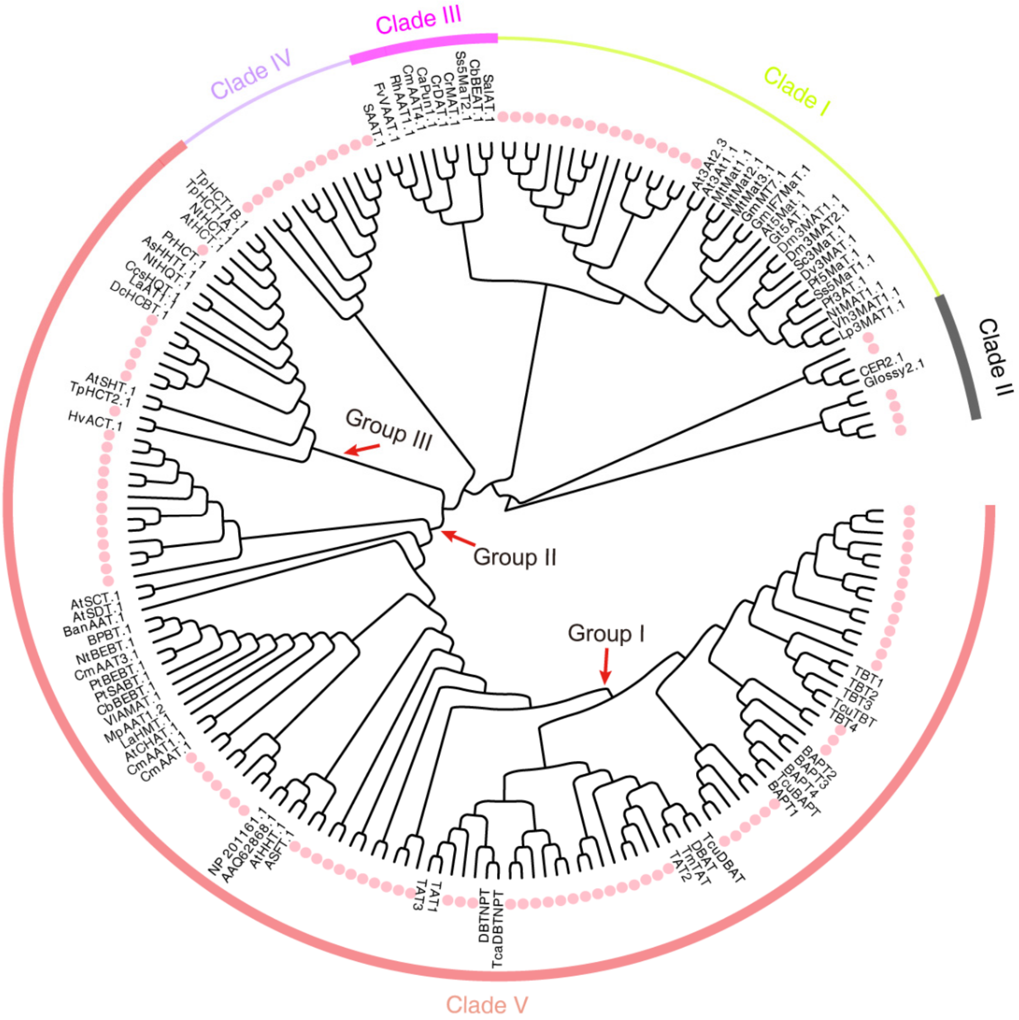

Additional Supplementary Fig.5 Phylogenetic analysis of Taxus BAHD acyltransferase proteins. The tree is generated using the neighbor-joining method and 1000 bootstraps based on putative amino acid full-length transferase sequences with MEGA 7.0 software and EvolView. The pink-red circle denotes transferase genes in *T. chinensis* var. *mairei.* The subgroups were designated based on a previous report[30]. All defined paclitaxel pathway BAHD acyltransferase genes are included in Group 1 of Clade V.

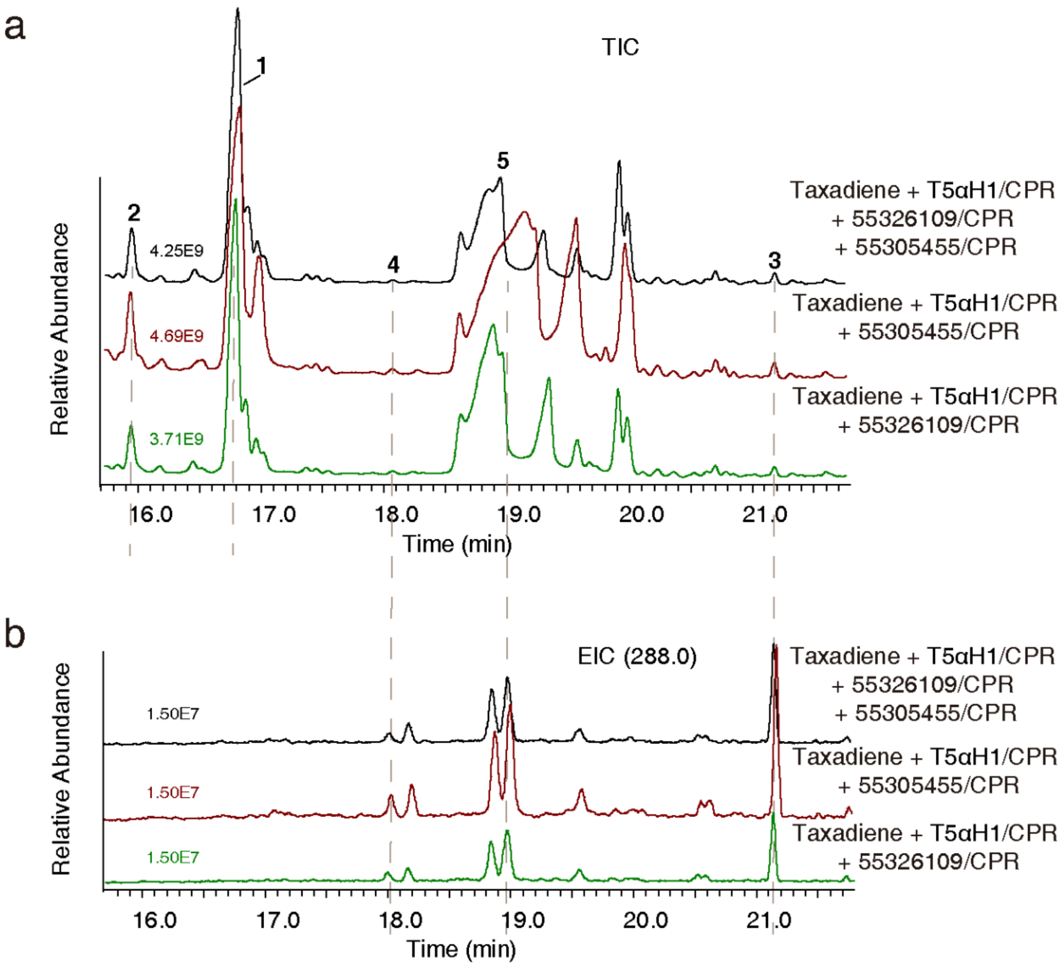

Additional Supplementary Fig.6 Enzymatic analysis of CYP725A 55326109 and 55305455 with taxane as the substrate. a, Total ion chromatograms (TICs) of the *in vitro* assays. Peaks 1-5 are the substrates taxa-4(5),11(12)-diene, taxa-4(20),11(12)-diene, taxa-4(20),11(12)-dien-5α-ol, OCT and iso-OCT, respectively. The taxadiene substrates (peaks **1** and **2**) were exogenously added, while the oxidized taxadienes (peaks **3**, **4,** and **5)** were from the T5αH/CPR catalytic products. **b,** Chromatograms of the extracted ions at 288 m/z^{+} for the oxidized taxadienes (peaks **3**, **4**, and **5**). There were no new peaks produced by the reaction mixtures, including T5αH/CPR and 55326109/CPR, T5αH/CPR and 55305455/CPR, and T5αH/CPR, and 55326109/CPR and 54359314/CPR.

3. Supplementary Tables

Supplementary Table 1 The Taxus genome assembly.

A. Sequencing data used to assemble the Taxus genome.

Material	Data Type	Size (Gb)
Endosperm	IIIumina (WGS)	693.73
Endosperm	Pacbio (CLR)	318.05
Leaf	Pacbio (HiFi)	26.06
Endosperm	Hi-C	1135.15
Total		2172.99

WGS, Whole-Genome Sequencing; CLR, Continuous Long Read; HiFi, High fidelity reads.

B. The statistics of the PacBio (CLR) assembly.

	Contig	Scaffold
Total	10,232,190,857 bp	10,237,952,243 bp
Count	15,880	3,813
Average	644,344.51 bp	2,685,012.39 bp
Max	21,889,323 bp	1,051,374,208 bp
N25	4,384,376 bp	957,407,293 bp
N50	2,436,244 bp	903,737,476 bp
N75	1,124,632 bp	769,507,007 bp
N90	426,395 bp	659,955,358 bp
N95	216,560 bp	411,570,604 bp

Supplementary Table 2 Statistics of Hi-C sequencing and mapping.

A. Raw data aligned to the genome.

Sample	Hi-C data
Clean Paired-end Reads	3,783,831,838
Unmapped Paired-end Reads	231,750,895
Unmapped Paired-end Reads Rate (%)	6.12
Paired-end Reads with Singleton	691,566,783
Paired-end Reads with Singleton Rate (%)	18.28
Multi Mapped Paired-end Reads	791,574,538
Multi Mapped Paired-end Reads Ratio (%)	20.92
Unique Mapped Paired-end Reads	2,068,939,622
Unique Mapped Paired-end Reads Ratio (%)	54.68

B. Valid data of reads aligned to the genome.

Sample	Hi-C data
Unique Mapped Paired-end Reads	2,068,939,622
Dangling End Paired-end Reads	47,562,609
Dangling End Paired-end Reads Rate (%)	2.30
Self Circle Paired-end Reads	5,581,545
Self Circle Paired-end Reads Rate (%)	0.27
Dumped Paired-end Reads	10,525,787
Dumped Paired-end Reads Rate (%)	0.51
Interaction Paired-end Reads	2,005,269,681
Interaction Paired-end Reads Rate (%)	96.92
Valid Paired-end Reads	2,001,492,791
Valid Paired-end Reads Rate (%)	96.74

Supplementary Table 3 The lengths of pseudochromosomes generated in the Hi-C assembly.

Psudochromosome	Length (bp)
Chr01	1,052,277,571
Chr02	995,865,175
Chr03	958,316,394
Chr04	950,298,798
Chr05	904,642,436
Chr06	909,132,773
Chr07	786,775,274
Chr08	774,176,834
Chr09	770,396,028
Chr10	681,618,719
Chr11	660,890,931
Chr12	412,525,553
Total anchored length	9,856,916,486
Unanchored length	381,035,757
Total length	10,237,952,243

Supplementary Table 4 Assembly and annotation statistics of the Taxus genome.

Parameter	Value
Genome size	10.23 Gb
GC content	36.78%
Contig number	15,879
N50 length (contig)	2.44 Mb
N90 length (contig)	426.40 kb
Longest contig	21,889,323 bp
Gene number	42,746
Average gene length	13,260 bp
Chromosomes	12
Exons per gene	3.5
Total anchored	9,856,916,486 bp
Repeat	76.09%
Anchored rate	96.28%

Supplementary Table 6 BUSCO analysis.

A. BUSCO analysis of genome annotation (embryophyte_odb10 (2020-9-10)).

	**Number**	**Rate (%)**
Complete BUSCOS (C)	1,052	65.2
Complete and single-copy BUSCOS (S)	980	60.7
Complete and duplicated BUSCOS (D)	72	4.5
Fragmented BUSCOs (F)	265	16.4
Missing BUSCOS (M)	297	18.4
Total BUSCO groups searched	1,614	100.0

B. BUSCO analysis of the gymnosperm species with genome data.

Species	Genome Length (Gb)	Contig N50 (kb)	C (%)	S (%)	D (%)	F (%)	M (%)
Pinus lambertiana[31]	31.0	246.6	73.0	67.9	5.1	7.1	19.9
Pinus taeda[32]	20.6	25.4	40.7	35.9	4.8	18.6	40.7
Picea abies[33]	19.6	4.9	27.4	24.0	3.4	25.8	46.8
Abies alba[34]	18.16	14.1	15.4	12.5	2.9	16.9	67.7
Pseudotsuga menziesii[35]	16.6	44.1	67.8	62.5	5.3	11.0	21.2
Ginkgo biloba[36]	10.61	48.2	69.4	61.3	8.1	10.3	20.3
Taxus chinensis var. *mairei*	9.9	2435.93	65.2	60.7	4.5	16.4	18.4
Sequoiadendron giganteum[37]	8.13	347.95	50.7	47.5	3.2	14.9	34.4
Gnetum montanum[38]	4.07	25.02	83.0	79.3	3.7	4.1	12.9

Abbreviation: C, Complete BUSCOs; S, Complete and single-copy BUSCOs; D, Complete and duplicated BUSCOs; F, Fragmented BUSCOs; M, Missing BUSCOs.

Supplementary Table 7 Functional annotation of Taxus genes.

Database	Number of annotated genes	Percentage (%)
GO	13,694	32.04
InterProScan	33,210	77.69
NR	32,802	76.74
Swiss-Port	28,093	65.72
TAIR	31,126	72.82
Total	36,518	85.43

Abbreviation: GO, Gene Ontology Database; NR, Non-Redundant Proteins; Swiss-Port, Swiss-Port Database. TAIR, The Arabidopsis Information Resource.

Supplementary Table 8 Statistics of repeated elements in the Taxus genome.

Classification	Number	Length (bp)	Percent of repeats (%)	Percent of genome (%)
Class I: Retroelement	2,712,885	4,219,248,359	54.16	41.21
LTR Retrotransposon	2,571,938	4,080,706,163	52.38	39.86
Ty1/Copia	473,313	504,043,663	6.47	4.92
Ty3/Gypsy	2,082,710	3,561,506,787	45.72	34.79
Other	15,915	15,155,713	0.19	0.15
Non-LTR Retrotransposon	140,947	138,542,196	1.78	1.35
LINE	140,871	138,531,871	1.78	1.35
SINE	76	10,325	0.00	0.00
Class II: DNA elements	299,098	283,535,871	3.64	2.77
CMC	7,686	1,266,320	0.02	0.01
hAT	81,774	69,108,347	0.89	0.68
PIF/Harbinger	219	124,131	0.00	0.00
MuLE-MuDR	135,519	158,064,978	2.03	1.54
Sola	19,436	25,932,250	0.33	0.25
Helitron	36,248	26,622,352	0.34	0.26
Enspm	17,812	2,384,418	0.03	0.02
Mariner	404	33,075	0.00	0.00
Small RNA	502	73,190	0.00	0.00
Simple repeats	53,662	26,564,977	0.34	0.26
Unknown	5,208,975	3,122,338,811	40.08	30.50
Total repeat fraction	8,416,069	7,790,303,404	100.00	76.09

Supplementary Table 27 Gene cluster identified through PlantiSMASH in the Taxus genome.

Type	Number	Chromosome
putative	9	1, 3, 9, 10
terpene	7	1, 3, 6, 8, 9
saccharide	13	2, 6, 8, 9, 10, 12
alkaloid	1	1
saccharide-polyketide	1	2
terpene-alkaloid	1	4
lignan-terpene	1	5
saccharide-terpene	1	10

4. Supplementary References 1-38

1 Li, Y. *et al.* Induction of half-sib embryonic callus and production of taxiod compounds from *Taxus chinensis* var. *mairei*. *Int. J. Agric. Biol.* **21**, 719-725, doi:10.17957/IJAB/15.0949 (2019).

2 Marcais, G. & Kingsford, C. A fast, lock-free approach for efficient parallel counting of occurrences of k-mers. *Bioinformatics* **27**, 764-770, doi:10.1093/bioinformatics/btr011 (2011).

3 Li, H. Minimap2: pairwise alignment for nucleotide sequences. *Bioinformatics* **34**, 3094-3100, doi:10.1093/bioinformatics/bty191 (2018).

4 Danecek, P. *et al.* Twelve years of SAMtools and BCFtools. *Gigascience* **10**, 1-4, doi:10.1093/gigascience/giab008 (2021).

5 Xu, Z. & Wang, H. LTR_FINDER: an efficient tool for the prediction of full-length LTR retrotransposons. *Nucleic Acids Res.* **35**, 265-268, doi:10.1093/nar/gkm286 (2007).

6 Ellinghaus, D., Kurtz, S. & Willhoeft, U. LTRharvest, an efficient and flexible software for de novo detection of LTR retrotransposons. *BMC Bioinform.* **9**, 18, doi:10.1186/1471-2105-9-18 (2008).

7 Ou, S. & Jiang, N. LTR_retriever: a highly accurate and sensitive program for identification of long terminal repeat retrotransposons. *Plant Physiol.* **176**, 1410-1422, doi:10.1104/pp.17.01310 (2018).

8 Potter, S. C. *et al.* HMMER web server: 2018 update. *Nucleic Acids Res.* **46**, 200-204, doi:10.1093/nar/gky448 (2018).

9 Edgar, R. C. MUSCLE: multiple sequence alignment with high accuracy and high throughput. *Nucleic Acids Res.* **32**, 1792-1797, doi:10.1093/nar/gkh340 (2004).

10 Arkin, A. P. FastTree 2 - approximately maximum-likelihood trees for large alignments. *PLoS One* **5**, e9490, doi:10.1371/journal.pone.0009490 (2010).

11 Keller, I., Bensasson, D. & Nichols, R. A. Transition-transversion bias is not universal: a counter example from grasshopper pseudogenes. *PLoS Genet.* **3**, e22, doi:10.1371/journal.pgen.0030022 (2007).

12 Rice, J., Longden, I. & Bleasby, A. EMBOSS: the European molecular biology open software suite. *Trends Genet.* **16**, 276-277, doi: 10.1016/s0168-9525(00)02024-2 (2000).

13 De La Torre, A. R., Li, Z., Van de Peer, Y. & Ingvarsson, P. K. Contrasting rates of molecular evolution and patterns of selection among gymnosperms and flowering plants. *Mol. Biol. Evol.*, 1363-1377, doi:10.1093/molbev/msx069 (2017).

14 Kim, D., Langmead, B. & Salzberg, S. L. HISAT: a fast spliced aligner with low memory requirements. *Nat. Methods* **12**, 357-360, doi:10.1038/nmeth.3317 (2015).

15 Pertea, M., Kim, D., Pertea, G. M., Leek, J. T. & Salzberg, S. L. Transcript-level expression analysis of RNA-seq experiments with HISAT, StringTie and Ballgown. *Nat. Protoc.* **11**, 1650-1667, doi:10.1038/nprot.2016.095 (2016).

16 Robinson, M. D., McCarthy, D. J. & Smyth, G. K. edgeR: a Bioconductor package for differential expression analysis of digital gene expression data. *Bioinformatics* **26**, 139-140, doi:10.1093/bioinformatics/btp616 (2009).

17 Wegrzyn, J. L., Lee, J. M., Tearse, B. R. & Neale, D. B. TreeGenes: a forest tree genome database. *Int. J. Plant Genomics* **2008**, 412875, doi:10.1155/2008/412875 (2008).

18 Altschul, S. F., Gish, W., Miller, W., Myers, E. W. & Lipman, D. J. Basic local alignment search tool. *J. Mol. Biol.* **215**, 403-410, doi:10.1016/S0022-2836(05)80360-2 (1990).

19 Li, L., Stoeckert, C. J., Jr. & Roos, D. S. OrthoMCL: identification of ortholog groups for eukaryotic genomes. *Genome Res.* **13**, 2178-2189, doi:10.1101/gr.1224503 (2003).

20 Edgar, R. MUSCLE: multiple sequence alignment with high accuracy and high throughput. *Nucleic Acids. Res.* **32**, 1792-1797, doi:10.1093/nar/gkh340 (2004).

21 Castresana, J. Selection of conserved blocks from multiple alignments for their use in phylogenetic analysis. *Mol. Biol. Evol.* **17**, 540-552, doi:10.1093/oxfordjournals.molbev.a026334 (2000).

22 Stamatakis, A. RAxML version 8: a tool for phylogenetic analysis and post-analysis of large phylogenies. *Bioinformatics* **30**, 1312-1313, doi:10.1093/bioinformatics/btu033 (2014).

23 Sanderson, M. J. r8s: inferring absolute rates of molecular evolution and divergence times in the absence of a molecular clock. *Bioinformatics* **19**, 301-302, doi:10.1093/bioinformatics/19.2.301 (2003).

24 De Bie, T., Cristianini, N., Demuth, J. P. & Hahn, M. W. CAFE: a computational tool for the study of gene family evolution. *Bioinformatics* **22**, 1269-1271, doi:10.1093/bioinformatics/btl097 (2006).

25 Yu, G., Wang, L. G., Han, Y. & He, Q. Y. clusterProfiler: an R package for comparing biological themes among gene clusters. *OMICS* **16**, 284-287, doi:10.1089/omi.2011.0118 (2012).

26 Urban, P., Mignotte, C., Kazmaier, M., Delorme, F. & Pompon, D. Cloning, yeast expression, and characterization of the coupling of two distantly related *Arabidopsis thaliana* NADPH-Cytochrome P450 reductases with P450 CYP73A5. *J. Biol. Chem.* **272**, 19176-19186, doi:10.1074/jbc.272.31.19176 (1997).

27 Pompon, D., Louerat, B., Bronine, A. & Urban, P. in *Methods in Enzymology* Vol. **272** (eds Eric F. Johnson & Michael R. Waterman) 51-64 (Academic Press, 1996).

28 Biggs, B. W. *et al.* Orthogonal assays clarify the oxidative biochemistry of taxol P450 CYP725A4. *ACS Chem. Biol.* **11**, 1445-1451, doi:10.1021/acschembio.5b00968 (2016).

29 Bian, G. *et al.* Production of taxadiene by engineering of mevalonate pathway in *Escherichia coli* and endophytic fungus *Alternaria alternata* TPF6. *Biotechnol. J.* **12**, doi:10.1002/biot.201600697 (2017).

30 Kuang, X., Sun, S., Wei, J., Li, Y. & Sun, C. Iso-Seq analysis of the *Taxus cuspidata* transcriptome reveals the complexity of Taxol biosynthesis. *BMC Plant Biol.* **19**, 210, doi:10.1186/s12870-019-1809-8 (2019).

31. Stevens, K. A. et al. Sequence of the sugar pine megagenome. *Genetics* **204**, 1613-1626, doi:10.1534/genetics.116.193227 (2016).

32. Zimin, A. V. et al. An improved assembly of the loblolly pine mega-genome using long-read single-molecule sequencing. *Gigascience* **6**, 1-4, doi: 10.1093/gigascience/giw016 (2017).

33. Nystedt, B. et al. The Norway spruce genome sequence and conifer genome evolution. *Nature* **497**, 579-584, doi:10.1038/nature12211 (2013).

34. Mosca, E. et al. A reference genome sequence for the european silver fir (*Abies alba* Mill.): A community-generated genomic resource. *G3* **9**, 2039-2049, doi: 10.1534/g3.119.400083 (2019).

35. Neale, D. B. et al. The Douglas-Fir genome sequence reveals specialization of the photosynthetic apparatus in Pinaceae. *G3* **7**, 3157-3167, doi:10.1534/g3.117.300078 (2017).

36 Guan, R. et al. Draft genome of the living fossil *Ginkgo biloba*. *Gigascience* **5**, 49, doi:10.1186/s13742-016-0154-1 (2016).

37 Scott, A. D. et al. A reference genome sequence for giant sequoia. *G3* **10**, 3907-3919, doi:10.1534/g3.120.401612 (2020).

38 Wan, T. et al. A genome for gnetophytes and early evolution of seed plants. *Nat. Plants* **4**, 82-89, doi:10.1038/s41477-017-0097-2 (2018).
