# Supplementary material for: The Taxus genome provides insights into paclitaxel biosynthesis
Source: Nat Plants. 2021 Jul 15;7(8):1026–36. doi: 10.1038/s41477-021-00963-5 (PMC8367818; doi:10.1038/s41477-021-00963-5)
Supplement: Supplementary file 2 — Reporting Summary [file 41477_2021_963_MOESM2_ESM.pdf]

Corresponding author(s): NPLANTS-201110158B

Last updated by author(s): May 31, 2021

Reporting Summary

Nature Research wishes to improve the reproducibility of the work that we publish. This form provides structure for consistency and transparency in reporting. For further information on Nature Research policies, see our Editorial Policies and the Editorial Policy Checklist.

Statistics

For all statistical analyses, confirm that the following items are present in the figure legend, table legend, main text, or Methods section.

n/a	Confirmed	
☐	☒	The exact sample size (*n*) for each experimental group/condition, given as a discrete number and unit of measurement
☐	☒	A statement on whether measurements were taken from distinct samples or whether the same sample was measured repeatedly
☐	☒	The statistical test(s) used AND whether they are one- or two-sided<br>*Only common tests should be described solely by name; describe more complex techniques in the Methods section.*
☒	☐	A description of all covariates tested
☒	☐	A description of any assumptions or corrections, such as tests of normality and adjustment for multiple comparisons
☐	☒	A full description of the statistical parameters including central tendency (e.g. means) or other basic estimates (e.g. regression coefficient) AND variation (e.g. standard deviation) or associated estimates of uncertainty (e.g. confidence intervals)
☐	☒	For null hypothesis testing, the test statistic (e.g. *F*, *t*, *r*) with confidence intervals, effect sizes, degrees of freedom and *P* value noted<br>*Give P values as exact values whenever suitable.*
☒	☐	For Bayesian analysis, information on the choice of priors and Markov chain Monte Carlo settings
☒	☐	For hierarchical and complex designs, identification of the appropriate level for tests and full reporting of outcomes
☒	☐	Estimates of effect sizes (e.g. Cohen's *d*, Pearson's *r*), indicating how they were calculated

Our web collection on statistics for biologists contains articles on many of the points above.

Software and code

Policy information about availability of computer code

Data collection	DNA-seq raw NGS data was generated by sequencing using the Illumina Hiseq 2500 platform. DNA-seq raw pacbio data were generated by sequencing using the Pacbio sequel II.<br>RNA-seq raw data were generated by sequencing using the Illumina Hiseq2500 platform.
Data analysis	All softwares used in the present study are publicly available and the corresponding software versions were described in detail in the Methods.<br>1.fastp v0.20.1 https://github.com/OpenGene/fastp<br>2.wtdbg2 v2.5 https://github.com/ruanjue/wtdbg2<br>3.GenomicConsensus v2.3.2 https://github.com/PacificBiosciences/GenomicConsensus<br>4.pilon v1.23 https://github.com/broadinstitute/pilon<br>5.Hi-C pro v2.11.1 https://github.com/nservant/HiC-Pro<br>6.juicer v1.6 https://github.com/aidenlab/juicer<br>7.3D-DNA v180922 https://github.com/aidenlab/3d-dna<br>8.TGS-Gapcloser v1.01 https://github.com/BGI-Qingdao/TGS-GapCloser<br>9.Purge Haplotigs v1.03 https://bitbucket.org/mroachawri/purge_haplotigs/src/master/<br>10.HISAT2 v2.1.0 http://daehwankimlab.github.io/hisat2/download/<br>11.StringTie v1.3.5 https://ccb.jhu.edu/software/stringtie/<br>12.RepeatModeler v1.0.1 http://www.repeatmasker.org/RepeatModeler/<br>13.RepeatMasker v4.0.5 http://repeatmasker.org/<br>14.LTR_finder v1.07 https://github.com/xzhub/LTR_Finder<br>15. genometools v1.5.10 http://genometools.org/pub/binary_distributions/<br>16.LTR_retriever v2.7 https://github.com/oushujun/LTR_retriever<br>17.SNAP v2013-02-16 https://github.com/KorfLab/SNAP<br>18.Augustus v3.2.2 https://github.com/Gaius-Augustus/Augustus

19.GlimmerHMM v3.0.4 http://ccb.jhu.edu/software/glimmerhmm/
20.Genewise v2.2.0 https://www.ebi.ac.uk/seqdb/confluence/display/THD/GeneWise
21.Tophat2 v.2.1.1 http://ccb.jhu.edu/software/tophat/index.shtml
22.Cufflinks v2.2.1 http://cole-trapnell-lab.github.io/cufflinks/
23.PASA v2.4.1 http://pasapipeline.github.io/
24.EVM v1.1.1 https://evidencemodeler.github.io/
25.ncbi-blast v2.2.26 ftp://ftp.ncbi.nlm.nih.gov/blast/executables/blast+/LATEST/
26.MCScanX v https://github.com/wyp1125/MCScanx
27.KaKs_Calculator v2.0 https://sourceforge.net/projects/kakscalculator2/
28. MEGA7 v7.0.26 https://www.megasoftware.net/dload_mac_beta
29. Plantismash v3.0.5 http://plantismash.secondarymetabolites.org/
30. edgeR R package v3.24.3 https://bioconductor.org/packages/release/bioc/html/edgeR.html
31. Jellyfish v2.1.3 https://github.com/gmarcais/Jellyfish
32. Sniffles v1.0.12 https://github.com/fritzsedlazeck/Sniffles/
33. MUSCLE v3.8.31 https://www.ebi.ac.uk/Tools/msa/muscle/
34. EMBOSS v6.6.0 http://emboss.sourceforge.net/
35. RAxML v8.2.9 https://cme.h-its.org/exelixis//web/software/raxml/index.html
36. Gblock v0.91b http://molevol.cmima.csic.es/castresana/Gblocks/Gblocks_documentation.html
37. orthoMCL v2.0.9 https://legacy.orthomcl.org/common/downloads/
38. r8s v1.81 https://sourceforge.net/projects/r8s/
39. cafe v3.0 https://github.com/hahnlab/CAFE
40. clusterProfiler R package v3.10.1 http://www.bioconductor.org/packages/release/bioc/html/clusterProfiler.html
41.pheatmap R package v1.0.12 https://www.rdocumentation.org/packages/pheatmap/versions/1.0.12
42. ggplot2 R package v3.3.3 https://cran.microsoft.com/web/packages/ggplot2/index.html
43. Gephi v0.92 https://gephi.org/
44. Pfam http://pfam.xfam.org/family/PF00067
45. Cytochrome P450 homepage http://drnelson.uthsc.edu/cytochromeP450.html
46. KAAS https://www.genome.jp/tools/kaas/
47. BUSCO v4.14 https://busco.ezlab.org/
48. Gene Ontology http://geneontology.org/
49. InterProscan v5.21-60.0 http://www.ebi.ac.uk/interpro/download/
50. Swiss-port https://www.uniprot.org/downloads
51. TrEMBL https://www.uniprot.org/downloads
52. TAIR https://www.arabidopsis.org/download/index-auto.jsp?dir=/download_files/Proteins

For manuscripts utilizing custom algorithms or software that are central to the research but not yet described in published literature, software must be made available to editors and reviewers. We strongly encourage code deposition in a community repository (e.g. GitHub). See the Nature Research guidelines for submitting code & software for further information.

Data

Policy information about availability of data

All manuscripts must include a data availability statement. This statement should provide the following information, where applicable:

- Accession codes, unique identifiers, or web links for publicly available datasets
- A list of figures that have associated raw data
- A description of any restrictions on data availability

The raw data used in this study have been deposited in the Genome Sequence Archive at the BIG Data Center, Beijing Institute of Genomics (BIG), Chinese Academy of Sciences and are accessible at http://bigd.big.ac.cn/gsa under bioproject PRJCA003841. The T. chinensis var. mairei genome sequences have been deposited at NCBI, under BioProject number PRJNA730337 and are publicly accessible at https://www.ncbi.nlm.nih.gov/Bioproject/?term=PRJNA730337.

Field-specific reporting

Please select the one below that is the best fit for your research. If you are not sure, read the appropriate sections before making your selection.

☒ Life sciences ☐ Behavioural & social sciences ☐ Ecological, evolutionary & environmental sciences

For a reference copy of the document with all sections, see nature.com/documents/nr-reporting-summary-flat.pdf

Life sciences study design

All studies must disclose on these points even when the disclosure is negative.

Sample size	Sample size was measured in a way that we are able to obtain statistically differences from at least three biological replicates. For RNA-seq, eight tissues from the female plant and the male plant and three half-sib cell lines were collected individually, at least three independent biological replicates were analyzed for each tissue. For MeJA treatment, 20-30 plates of Taxus cell lines were used for each assay.
Data exclusions	We excluded Taxus cell lines that displayed growth defects prior to treatment.
Replication	For measurements of RNA-seq, qRT-PCR, taxane metabolite, kinetic assay, and TS in vivo assay at least three independent measurements were statistically analyzed.

Randomization	In the present study, the male and female of Taxus plants and cell lines of Taxus were used as the materials. The plants were grown in the field, and cell lines were cultivated in growth chambers. All the materials used for experiments were randomly selected.
Blinding	Experiments was blinded and carried out by different coauthors or other researchers.

Reporting for specific materials, systems and methods

We require information from authors about some types of materials, experimental systems and methods used in many studies. Here, indicate whether each material, system or method listed is relevant to your study. If you are not sure if a list item applies to your research, read the appropriate section before selecting a response.

Materials & experimental systems

n/a	Involved in the study
☐	☒ Antibodies
☐	☒ Eukaryotic cell lines
☒	☐ Palaeontology and archaeology
☒	☐ Animals and other organisms
☒	☐ Human research participants
☒	☐ Clinical data
☒	☐ Dual use research of concern

Methods

n/a	Involved in the study
☒	☐ ChIP-seq
☒	☐ Flow cytometry
☒	☐ MRI-based neuroimaging

Antibodies

Antibodies used	Flag Tag, Beyotime, China, Cat#AF5051, Flag Tag Mouse Monoclonal Antibody, 1:1000 dilution in vitro;<br>IgG(H+L), Beyotime, China, Cat#A0216， Sheep Polyclonal Antibody Conjugated with Horseradish Peroxidase, 1:1000 dilution in vitro.
Validation	The mouse monoclonal antibody for the indicated epitope tags and sheep polyclonal antibody conjugated with horseradish peroxidase (Beyotime, Cat#AF5051 and A0216) were validated by the respective company (Beyotime: https://www.beyotime.com/index.htm). These antibodies are commonly used in molecular biology research.

Eukaryotic cell lines

Policy information about cell lines

Cell line source(s)	The homozygous haploid callus and two half-sib cell lines (HC, a high paclitaxel-yielding cell line; LC, a low paclitaxel-yielding line) were induced from endosperm of Taxus chinensis var. mairei by ourself.
Authentication	These Taxus cell lines are published in plant science-related studies (doi:10.1186/s40064-016-2320-4, doi:10.17957/IJAB/15.0949).
Mycoplasma contamination	All Taxus cell lines were tested regularly for Mycoplasma contamination.
Commonly misidentified lines<br>(See ICLAC register)	No commonly misidentified cell lines were used.
