# Supplementary figures and images for: The Taxus genome provides insights into paclitaxel biosynthesis
Source: Nat Plants. 2021 Jul 15;7(8):1026–36. doi: 10.1038/s41477-021-00963-5 (PMC8367818; doi:10.1038/s41477-021-00963-5)

## Additional Supplementary Fig.4c

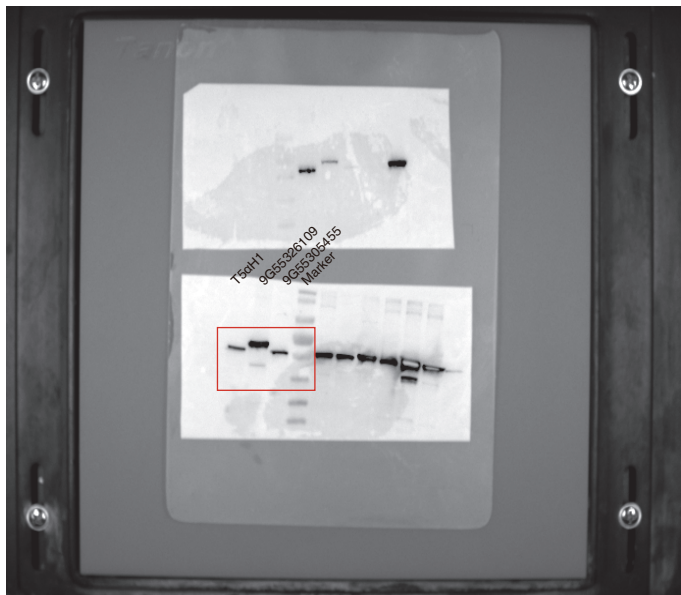

Unprocessed gel scan file

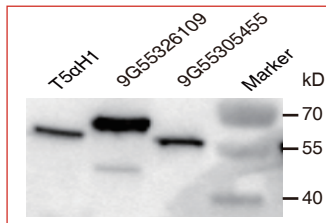

The gel figure on display

Supplement: Supplementary file 4 — Unprocessed western blots for Supplementary Fig. 4. These images were collected from the TANON 5200 Automatic Chemiluminescence Imaging Analysis System. [file 41477_2021_963_MOESM4_ESM.pdf]
